# Distinct Patterns of Selective Sweep and Polygenic Adaptation in Evolve and Resequence Studies

**DOI:** 10.1093/gbe/evaa073

**Published:** 2020-04-18

**Authors:** Neda Barghi, Christian Schlötterer

**Affiliations:** Institut für Populationsgenetik, Vetmeduni, Vienna, Austria

**Keywords:** polygenic adaptation, selective sweep model, trait optimum model, quantitative trait, laboratory natural selection, computer simulations

## Abstract

In molecular population genetics, adaptation is typically thought to occur via selective sweeps, where targets of selection have independent effects on the phenotype and rise to fixation, whereas in quantitative genetics, many loci contribute to the phenotype and subtle frequency changes occur at many loci during polygenic adaptation. The sweep model makes specific predictions about frequency changes of beneficial alleles and many test statistics have been developed to detect such selection signatures. Despite polygenic adaptation is probably the prevalent mode of adaptation, because of the traditional focus on the phenotype, we are lacking a solid understanding of the similarities and differences of selection signatures under the two models. Recent theoretical and empirical studies have shown that both selective sweep and polygenic adaptation models could result in a sweep-like genomic signature; therefore, additional criteria are needed to distinguish the two models. With replicated populations and time series data, experimental evolution studies have the potential to identify the underlying model of adaptation. Using the framework of experimental evolution, we performed computer simulations to study the pattern of selected alleles for two models: 1) adaptation of a trait via independent beneficial mutations that are conditioned for fixation, that is, selective sweep model and 2) trait optimum model (polygenic adaptation), that is adaptation of a quantitative trait under stabilizing selection after a sudden shift in trait optimum. We identify several distinct patterns of selective sweep and trait optimum models in populations of different sizes. These features could provide the foundation for development of quantitative approaches to differentiate the two models.

## Introduction

Characterizing adaptive traits and, more recently, identification of their genetic basis has been one of the long-standing research fields in evolutionary biology. The fields of molecular population genetics and quantitative genetics have had different approaches in addressing the genetic basis of phenotypic adaptation. Molecular population genetic theory assumes that adaptation occurs via independent mutations that rise in frequency until fixation (Maynard Smith and Haigh 1974), that is, hard sweeps. Therefore, adaptation is viewed to occur by large frequency changes of beneficial mutations. More recently, the concept of classic hard sweeps has been extended by soft sweeps—the beneficial allele either starts from standing genetic variation or multiple beneficial alleles are generated by mutation in the same gene (Hermisson and Pennings 2005; Przeworski et al. 2005). Beneficial alleles may not be fixed quickly; under weak selection, allele frequency changes (AFCs) will be small and they may only become fixed after a very long time. For decades, the selective sweep model has dominated molecular population genetics and the distortion of the allele frequency spectrum of sites flanking beneficial mutations has been exploited by a wealth of statistical tests to distinguish selective sweeps (hard and soft) from neutrality (e.g., in Messer and Petrov 2013; Pavlidis and Alachiotis 2017). Many traits such as HIV resistance, lactose tolerance, and pesticide resistance show molecular signatures compatible with hard and soft selective sweeps (reviewed by Messer and Petrov [2013]).

Quantitative genetics, on the other hand, traditionally has a strong focus on the evolution of phenotype, which is assumed to be determined by many contributing alleles, each with subtle effect, that is, infinitesimal model (Barton et al. 2017). Therefore, the AFC caused by selection on each locus is very small. Although evolution of adaptive traits frequently seems to be influenced by many loci (Burke et al. 2010; Turner et al. 2011; Tobler et al. 2014; Barghi et al. 2019), only recently the genomic signature of polygenic adaptation is being studied (Chevin and Hospital 2008; Pritchard and Di Rienzo 2010; Pritchard et al. 2010; Jain and Stephan 2017; Höllinger et al. 2019). Statistical tests for detecting polygenic adaptation all rely on the collective response of many loci, each displaying only small AFCs (Turchin et al. 2012; Berg and Coop 2014; Field et al. 2016). These methods have identified the mode of adaptation to be polygenic for many traits such as height in human populations (Berg and Coop 2014), body size in Atlantic silverside (Therkildsen et al. 2019), and morphological features in sticklebacks (Conte et al. 2015).

The main difference between selective sweep and polygenic adaptation is that AFC of each locus is not influenced by other loci under the selective sweep model, whereas selection in polygenic adaptation is collective and loci interact epistatically for fitness. Subtle frequency changes can be observed for selective sweeps (Höllinger et al. 2019). On the other hand, recent theoretical (Chevin and Hospital 2008; Jain and Stephan 2017; Hayward and Sella 2019; Höllinger et al. 2019; Thornton 2019) and empirical studies (Barghi et al. 2019) demonstrated that polygenic adaptation can also occur by the means of large allelic frequency changes (sweep-like selection signatures). Sweep-like selection signatures arising from selective sweeps and polygenic adaptation suggest that the standard approach of studying genomic signatures in extant/evolved populations is not conclusive about the underlying model. Because knowledge of the underlying model of adaptation is crucial for the proper theoretical modeling and neutrality tests, alternative approaches are needed to distinguish between them and determine their importance for adaptation processes.

Time series data provide information about the trajectories of beneficial alleles in evolving populations, which can be used to distinguish between the models. Time series data are, however, quite rare. In addition to fossil data, experimental evolution provides a powerful approach to study the adaptive architecture of traits (Kawecki et al. 2012; Schlötterer et al. 2015). The cost-effectiveness of sequencing pools of individuals (Schlötterer et al. 2014) provides the opportunity to generate time series of genome-wide polymorphism data in multiple replicates.

Recently, the extent of genomic similarity among replicates was used as a summary statistic to determine the underlying evolutionary model in ten experimental replicates of *Drosophila simulans* (Barghi et al. 2019). With a single discriminating summary statistic not being powerful enough, in this study we aim to identify additional patterns in the evolving populations which are informative for recognizing the underlying evolutionary model.

Reasoning that genetic drift provides a major perturbation of the directed forces of selection, we explored the potential of different experimental population sizes to distinguish between the selective sweep and polygenic models. Here, we define polygenic adaptation as “trait optimum model” that is adaptation of a quantitative trait under stabilizing selection after a sudden shift in trait optimum via alleles that interact epistatically for fitness. Selective sweep model is adaptation of a trait via independent beneficial mutations that are conditioned for fixation. Using computer simulations, we identify several parameters such as allele frequency trajectories, time series fitness, distribution of selected alleles on haplotypes, and parallelism among replicates that distinguish sweep and trait optimum models.

## Materials and Methods

We simulated a quantitative trait with linked loci under sweep and trait optimum models for two population sizes, that is, 450 and 9,000 diploid individuals, assuming random mating among individuals (scenario A in table 1*a* and *b*). We define the trait optimum model as polygenic adaptation of a quantitative trait after a shift in phenotypic optimum. The positions of the selection targets were randomly distributed along the entire chromosomes 2 and 3 of *D. simulans* but kept the same for sweep and trait optimum models. For a realistic linkage structure and to mimic the number of haplotypes typically used in evolve and resequence (E&R) studies, we used 189 haplotypes from a *D. simulans* population collected in Florida (Howie et al. 2019) to construct populations of 450 and 9,000 individuals for the simulations, that is, each haplotype is present in multiple copies in the founder population. We used the recombination landscape of *D. simulans* in our simulations (Howie et al. 2019). Population fitness (sweep model) or phenotype (trait optimum model) and allele frequencies were recorded every tenth generation until generation 140. Each simulation scenario was performed in 500 iterations. For characterization of the qualitative differences between sweep and trait optimum model, we performed computer simulations using functions *w* (sweep) and *qff* (trait optimum) of MimicrEE2 (version mim2-v193) (Vlachos and Kofler 2018).

**Table 1:** Simulation Parameters for Sweep and Trait Optimum Models

(*a*) Sweep			
Model	A	B	C
	Default	Different no. of loci	Different *s*
Parameters			
*N*	450, 9,000	450, 9,000	450, 9,000
No. of loci	100	10, 20, 50, 100	100
Selection	0.08	0.08	0.02, 0.05, 0.08, 0.1
Starting frequency	0.05	0.05	0.05
Recombination map	*Drosophila simulans*	*Drosophila simulans*	*Drosophila simulans*

**(*b*) Trait optimum**			
**Model**	**A**	**B**	**C**
	**Default**	**Different no. of loci**	**Different effect size**

Parameters			
*N*	450, 9,000	450, 9,000	450, 9,000
No. of loci	100	10, 20, 50, 100	100
Effect size	0.04	0.04	0.04, 0.08, 0.2, 0.4
Fitness function	Gaussian fitness function with standard deviation of 0.3, fitness ranges between 0.5 and 4.5. Optimum phenotype is −2.5	Gaussian fitness function with standard deviation of 0.3, fitness ranges between 0.5 and 4.5. Optimum phenotype varies depending on the no. of loci^a^	Gaussian fitness function with standard deviation of 0.3, fitness ranges between 0.5 and 4.5. Optimum phenotype varies depending on the effect size of loci^b^
Starting frequency	0.05	0.05	0.05
Heritability	0.5	0.5	0.5
Recombination map	*Drosophila simulans*	*Drosophila simulans*	*Drosophila simulans*

^a^ Fitness functions are shown in supplementary figure S13*A*, Supplementary Material online.

^b^ Fitness functions are shown in supplementary figure S13*B*, Supplementary Material online.

### Simulations of Selective Sweep Model

We performed forward Wright–Fisher simulations using 100 linked loci (linkage structure of the phased haplotypes [Howie et al. 2019]) with equal starting frequencies of 0.05 and equal selection coefficients of 0.08 constant across time in populations of 450 and 9,000 diploid individuals for 140 generations (scenario A in table 1*a*). In addition to this default scenario, we also performed simulations with different numbers of contributing loci, for example, 10, 20, 50, and 100 (scenario B in table 1*a*) and different values for the selection coefficient, for example, 0.02, 0.05, 0.08, and 0.1 (scenario C in table 1*a*) in populations of 450 and 9,000 diploid individuals.

### Simulations of Trait Optimum Model

In trait optimum simulations, we simulated adaptation of a quantitative trait to a new trait optimum. Trait *z* is affected by *L* diallelic loci. The effect size of the “+” allele is +*a* with frequency *p_i_* and the effect size of the “−” allele is –*a* with frequency *q_i_* = 1 − *p_i_*. Trait *z* is computed as

(1) $$\;z=\sum_{i=1}^{L}a_{i}\left(p_{i}-q_{i}\right)+2p_{i}q_{i}d_{i}.$$

We assume codominance (*h* = 0.5), *d *=* *0, and epistasis is neglected, thus trait *z* was determined additively. The trait value is mapped to fitness (*w*) using a Gaussian fitness function where PDF is the probability distribution function and max*fit* and min*fit* are the maximum and minimum fitness values:

(2) $$\mathrm{PDF}=\;\frac{1}{\sqrt{2\pi {\left(sd\right)}^{2}}}\times e^{-\frac{{\left(x-\mu \right)}^{2}}{2{\left(sd\right)}^{2}}},$$

(3) $$\mathrm{fitness}\;\left(w\right)=\mathrm{PDF}\frac{\max\mathrm{fit}-\min\mathrm{fit}}{\max\left(\mathrm{PDF}\right)}+\min\mathrm{fit}.$$

We simulated 100 linked loci starting at frequency of 0.05 with equal effects, that is, 0.04 in populations of 450 and 9,000 diploid individuals (scenario A in table 1*b*). The trait optimum (phenotype) was set at −2.5 (*µ*) with standard deviation of 0.3 and fitness ranged between 0.5 and 4.5 (scenario A in table 1*b*). In addition, further simulations with different number of loci (scenario B in table 1*b*) and different values for effect sizes (scenario C in table 1*b*) were performed; for each simulation run, the same effect sizes were used for all loci. The phenotypic value of the populations at the beginning of the simulations varies depending on the effect size and the number of loci. To enable comparison of simulations with different number of contributing loci and/or different effect sizes, independent of the phenotypic variance in the founder population, we adjusted the phenotypic optimum for each simulation scenario such that all populations move the same distance in the phenotypic space to reach the phenotypic optimum (supplementary fig. S13, Supplementary Material online).

### Neutral Simulations

To account for the effect of drift in AFCs, we performed simulations for populations with 450 and 9,000 individuals with no selection; all parameters of simulations matched scenario A in table 1*a* but without selection. We determined the AFCs, and set the threshold for identification of alleles with AFC more than expected under drift based on the upper 5% tail of neutral AFC distribution between the founder and evolving populations at each time point across 500 iterations (supplementary fig. S1, Supplementary Material online).

### Repeatability of Adaptation (Similarity among Replicates)

The average pairwise Jaccard indices (Jaccard 1901) among the replicates were calculated for 50 sets of ten-replicate populations of the sweep and trait optimum simulations using the number of alleles with AFCs more than expected under drift (using the neutral simulations above).

## Results and Discussion

With recent theoretical (Chevin and Hospital 2008; Jain and Stephan 2017; Höllinger et al. 2019) and empirical studies (Barghi et al. 2019) demonstrating that polygenic adaptation can also result in sweep-like selection signatures, it has become clear that the distinction of the underlying selection model requires new approaches building on multiple diagnostic features. For example, we recently showed that evolutionary models can be distinguished by the extent to which targets of selection are shared among replicates (Barghi et al. 2019). However, a reliable distinction between models requires identification of additional features that distinguish models. We performed computer simulations under sweep and trait optimum models with small and large populations sizes to identify distinct patterns for each model. Our computer simulations are not designed to exhaustively cover all possible parameter combinations, but we rather identify distinct features of each model.

### Distinct Characteristics of Sweep and Trait Optimum Models

We explored potential differences between selective sweep and trait optimum models using a standard set of simulation parameters. In a population of 450 diploid individuals, 100 linked beneficial loci, matching typical E&R experiments in *Drosophila* (Barghi et al. 2019), were simulated. Beneficial alleles had the same starting frequency of 0.05 and equal effects (selection coefficient of 0.08 for selective sweep and effect size of 0.04 for trait optimum model). Simulations were performed using the *D. simulans* recombination landscape (Howie et al. 2019) in 500 iterations (scenario A in table 1*a*, for sweep, and table 1*b*, for trait optimum model) using MimicrEE2 (Vlachos and Kofler 2018).

Typical E&R studies have relatively small population sizes, which require accounting for the expected AFC due to genetic drift to distinguish selection from neutrality. Regardless of the selection model, genetic drift is quite strong in small populations (supplementary fig. S1, Supplementary Material online). We accounted for this by computing a frequency cutoff based on the 95% quantile of AFC under neutral simulations and only alleles with more extreme AFCs were considered to be selected (supplementary fig. S1, Supplementary Material online).

#### Allele Frequency Trajectories

**Fig. 1.**
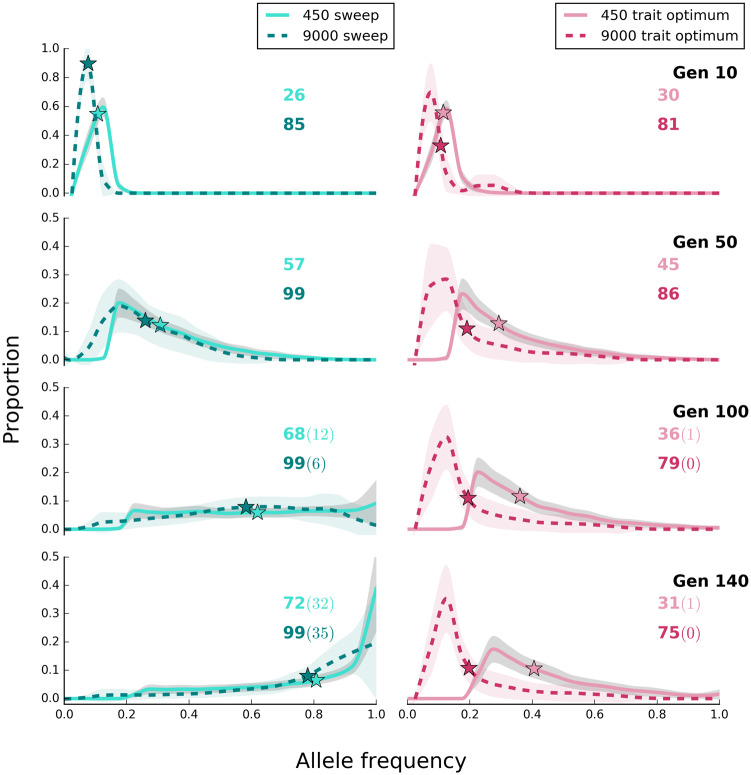
—The site frequency spectrum of selected alleles differs in large and small populations for the sweep and trait optimum model. Populations with 450 and 9,000 individuals evolved for 140 generations under the two different selection regimes (scenario A in table 1*a* and *b*). The lines show the average (binned from 0 to 1 with 0.05 intervals) frequency of selected alleles across 500 replicates and shaded areas depict standard deviation. On the *y* axis (proportion), we show the fraction of loci that experienced a larger frequency increase than expected under neutrality. Asterisks depict the median frequency increase of selected alleles averaged across 500 replicates. The number of alleles with frequency increase averaged across 500 replicates is shown with colors corresponding to the labels. The number of alleles with sweep-like signature (frequency ≥ 0.9) averaged across 500 replicates, if present, is shown in parentheses. Rows correspond to time points of the experiments, that is, generation, and shown as “Gen #.” The site frequency spectra of selected alleles in ten generation intervals are presented in supplementary figure S2, Supplementary Material online.

**Fig. 2.**
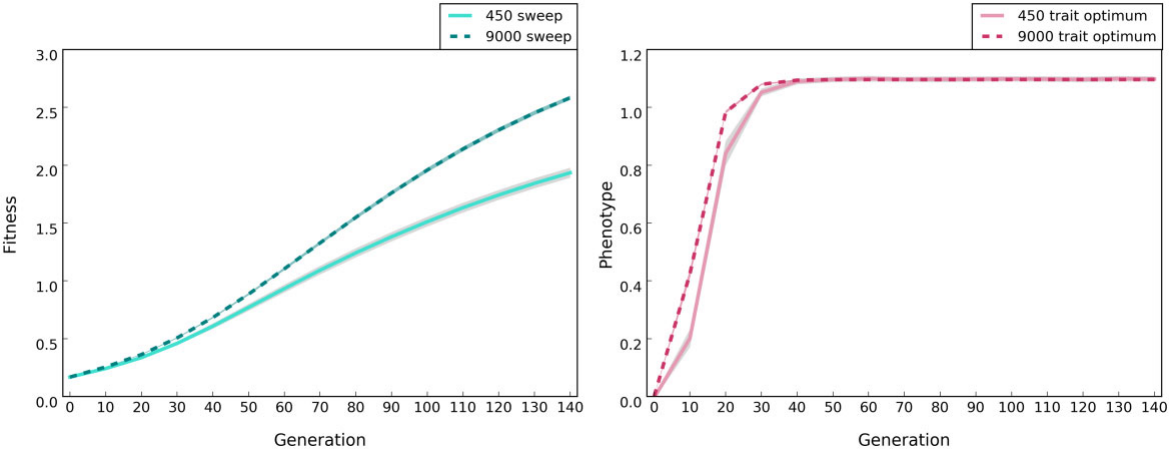
—Distinct pattern of fitness and phenotype evolution under sweep and trait optimum models, respectively. Populations of 450 (solid lines) and 9,000 (dotted lines) individuals were simulated (scenario A in table 1*a* and *b*). Lines depict the median fitness or phenotype averaged across 500 replicates and the shaded areas show standard deviation. Fitness is log_10_ transformed. The optimum phenotype in trait optimum model (right panel) is 1.1. For plotting the phenotype across different founder populations, we normalized it by subtracting the average phenotype of the population at generation 0 from the phenotype of every individual at each time point.

One important difference between the two models is the pattern of AFCs. Under the selective sweep model, selected alleles continuously increase in frequency until they reach fixation (fig. 1, solid green lines), whereas distinct phases of AFCs were discerned for the trait optimum model (Franssen et al. 2017). In the initial phase of adaptation, when the population is far from the trait optimum, most alleles increase in frequency (fig. 1, solid red lines). After the phenotypic optimum is reached (around generation 40, as seen in fig. 2), the second phase starts where the allele frequencies plateau (that is, the allele frequencies do not change much anymore, fig. 1, dotted red lines). However, drift affects this phase and in small populations this phase is either very short or not present at all. In small populations, drift decreases the frequency of some alleles below the threshold for identification of selected alleles, and with loss of these alleles, the median frequency of the remaining alleles continues to rise (fig. 1, solid red line). The third phase of AFCs includes fixation and loss of selected alleles. The first two phases are shown in figure 1 (for more time points, see supplementary fig. S2, Supplementary Material online); the third phase becomes noticeable after more generations, for example, 2,500 (supplementary fig. S3, Supplementary Material online). We illustrate the expected allele frequencies under sweep model and the first two phases of the trait optimum model by showing the trajectories of alleles in a single replicate in supplementary figure S4, Supplementary Material online.

#### Sweep-Like Signatures

Many alleles reach frequency of ≥0.9, that is, they exhibit sweep-like signatures, in the sweep model (generation 60 onward in small populations, fig. 1, solid green lines), whereas such signatures are typically not observed in the trait optimum model (fig. 1, solid red lines).

#### Fitness

As the frequency of selected alleles rises under the selective sweep model (fig. 1, solid green lines), population fitness also increases until all selected alleles are fixed (fig. 2, solid green line). Unlike the sweep model, the population fitness under the trait optimum model increases only until the phenotypic optimum is reached (fig. 2, solid red line). One distinct feature of the two models is that for sweep model the phenotypic value increases as long as the frequency of selected alleles does so. For the trait optimum model, AFCs (fig. 1) are decoupled from the phenotype as soon as the trait optimum has been reached (fig. 2).

#### Parallelism across Replicates

**Fig. 3.**
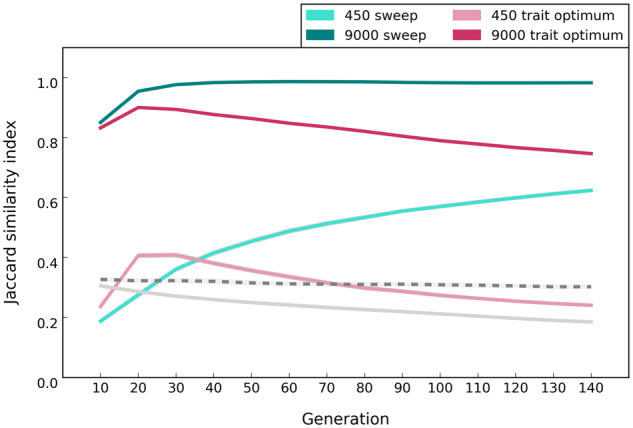
—Distinctive patterns of parallelism (that is, similarity among replicate populations) under sweep and trait optimum models in populations of 450 and 9,000 individuals (scenario A in table 1*a* and *b*). We used the Jaccard similarity index, which quantifies the extent to which alleles are shared among replicates (0 = no overlap, 1 = complete sharing), to quantify the similarity among replicate populations. Lines show the average pairwise Jaccard indices among replicates for 50 sets of ten-replicate evolution experiments and the shaded area around each line shows standard deviation. Solid and dotted gray lines show the average Jaccard index under neutrality in populations of 450 and 9,000 individuals, respectively, and standard deviation is shown as the shaded area around each line. For the trait optimum model, the optimum phenotype is reached at generation 40 and 30 in small and large populations, respectively (as seen in fig. 2, right panel).

We use the number of selected alleles shared among replicates as a measure for parallelism. Because the loss of alleles is more common in small populations due to drift, different selected alleles may be detected among replicates resulting in lower parallelism among replicates (fig. 3). This feature is shared between the two models. For the sweep model, parallelism continues to increase as more alleles reach frequencies above neutrality. In trait optimum model, the loci have epistasis for fitness and thus genetic redundancy is an intrinsic feature of the model. Genetic redundancy describes the phenomenon that more alleles are segregating in a population than needed to reach the trait optimum (Goldstein and Holsinger 1992; Nowak et al. 1997; Yeaman 2015; Barghi et al. 2019). In this case, if some alleles contributing to the phenotype are lost, the trait optimum can still be reached by frequency increase of the remaining alleles. In the trait optimum model, parallelism increases until populations reach the phenotypic optimum but it decreases afterward (fig. 3). This pattern can be explained by some alleles decreasing their frequency below the detection cutoff after the trait optimum is reached (for small population from generation 40 in fig. 1). Because the stochasticity of the small populations in the first phase results in different loci contributing to the reach of trait optimum, the loss of alleles due to stochasticity in the second phase reduces the parallelism even more (fig. 3). For small populations, the similarity among replicates is higher than for neutral populations when neutral alleles are used to calculate the Jaccard index, but the difference becomes rather small after the trait optimum has been reached due to the large influence of genetic drift.

#### Distribution of Selected Alleles on Haplotypes

**Fig. 4.**
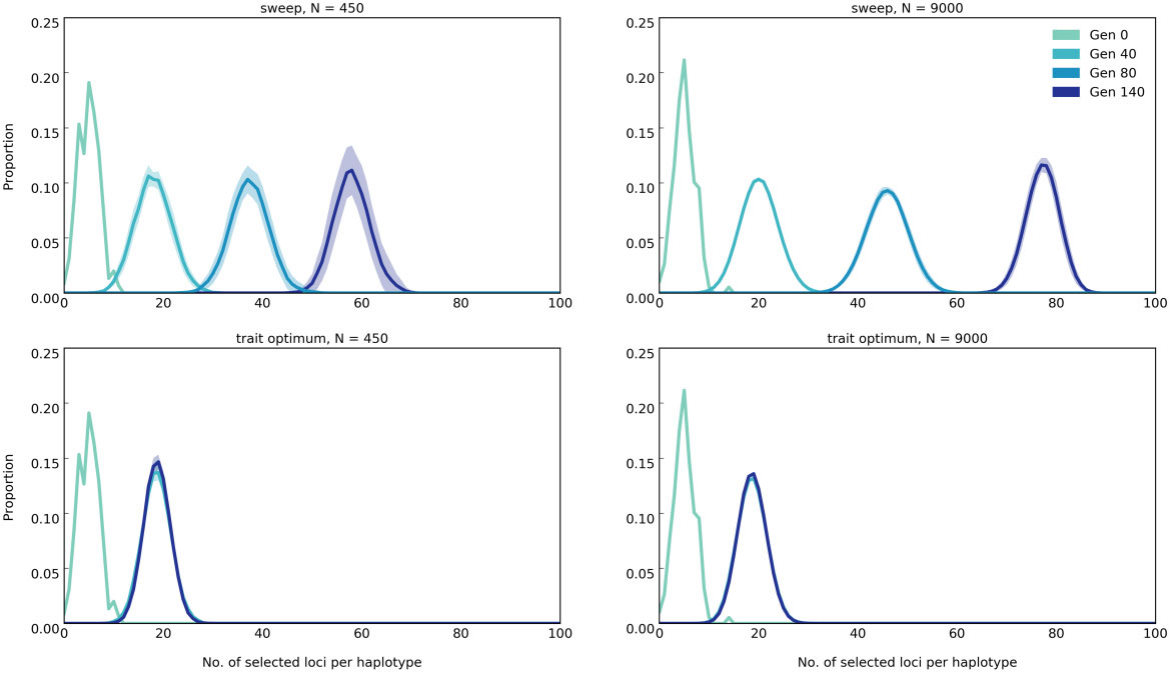
—The distribution of number of beneficial loci per haplotype under sweep (top panels) and trait optimum (bottom panels) models in populations of 450 and 9,000 individuals (scenario A in table 1*a* and *b*). Lines show the number of beneficial loci per haplotype (binned from 0 to 100 with intervals of 1) averaged across 50 replicates and the shaded area around lines show standard deviation. On the *y* axis (proportion), we show the fraction of haplotypes with specific number of beneficial loci from the total number of haplotypes in each replicate. Time points, that is, generation, are shown as “Gen #.”

In our simulations, the beneficial alleles were randomly distributed across the chromosomes in the founder populations so that each haplotype carries on average five to six beneficial alleles (fig. 4). Due to recombination, the number of beneficial alleles per haplotype increases in both models. Although under the sweep model, the number of beneficial loci per haplotype continues to increase until fixation of all alleles (fig. 4), for the trait optimum model, this number increases only until the fitness optimum is reached (at generation 40 for small populations, as seen in fig. 2) but does not change afterward. Thus, another distinctive pattern between the two models is the plateau in the number of beneficial alleles per haplotype in the trait optimum model, whereas this number continuously increases under the sweep model until all alleles are fixed.

### Effect of Population Size

Large populations experience less genetic drift than small ones, which increases the efficacy of selection and the power to detect selected alleles. To assess the impact of population size on the ability to discriminate between sweep and trait optimum models, we also performed simulations with a larger population size, that is, 9,000 diploid individuals (scenario A in table 1*a*, for sweep, and table 1*b*, for trait optimum model). Comparison of the sweep and trait optimum models in small and large populations revealed additional distinctive features to differ between the models possible only by the combined analysis of different population sizes.

#### Allele Frequency Changes

The 95% quantile AFC under neutrality in the large population (only 0.034 until generation 140, supplementary fig. S1, Supplementary Material online) is much less than in small populations (0.18, supplementary fig. S1, Supplementary Material online). Therefore, a plateau of the median allele frequencies after reaching the optimal trait under the trait optimum model is observed in large populations (fig. 1, dotted red lines) which provides an unambiguous signature differentiating the two models.

The difference in median allele frequencies between small and large populations increases with time for the trait optimum model (fig. 1). This pattern is the consequence of more loci decreasing below the detection limit in the small populations than for large ones after the trait optimum has been reached. For the sweep model, the allele frequencies continuously increase with time so the slight difference in the median allele frequencies between small and large populations decreases continuously. Hence, large and small populations have characteristic signatures that distinguish trait optimum model from sweep model. Combining the information from large and small populations provides an even stronger distinction between the two models.

#### Fitness

The evolution of fitness has the same trend in small and large populations regardless of the evolutionary model. The increase in fitness is higher in large populations than in the small ones in the sweep model (fig. 2) because fewer alleles are lost by drift (fig. 1). Furthermore, the population fitness increases faster in the large population under the trait optimum model but only until the phenotypic optimum is reached (fig. 2). Despite faster increase of fitness in large populations under trait optimum model, small and large populations reach the fitness optimum almost at the same time (fig. 2) and the differences in fitness between small and large populations before reaching the fitness optimum are very subtle. However, in the sweep model, the difference in fitness gain between small and large populations increases with time. Thus, the differential fitness in populations of different sizes can serve as discriminator between the two models.

#### Parallelism across Replicates

Regardless of the evolutionary model, the signature of selection is more repeatable in large populations than in small ones because fewer alleles are lost due to drift (fig. 3). In large populations, the similarity among replicates, measured by the Jaccard index, is considerably higher than for neutral alleles. In small populations the difference between neutral and beneficial alleles is less pronounced.

#### Distribution of Selected Alleles on Haplotypes

Under the sweep model, more selected alleles are recombined onto the same haplotype in large populations than in small ones (fig. 4) and the number of selected alleles increases with time for both population sizes. For the trait optimum model, population size has no influence on the number of selected alleles on haplotypes after trait optimum is reached (fig. 4).

Although for some discriminatory features, such as distribution of selected alleles on haplotypes, no major difference can be noted between large and small populations, contrasting the patterns of fitness evolution, AFCs and parallelism among replicates in small and large populations clearly provides some additional information not available from analysis of a single population size alone.

### Effect of the Number of Selection Targets

We determined the influence of the number of beneficial alleles by simulating 10, 20, 50, and 100 linked loci (scenario B in table 1*a*, for sweep, and table 1*b*, for trait optimum model) with starting frequency of 0.05 and equal effects (0.08 for selective sweep and 0.04 for trait optimum model) in small (450) and large (9,000) populations in 500 iterations.

**Fig. 5.**
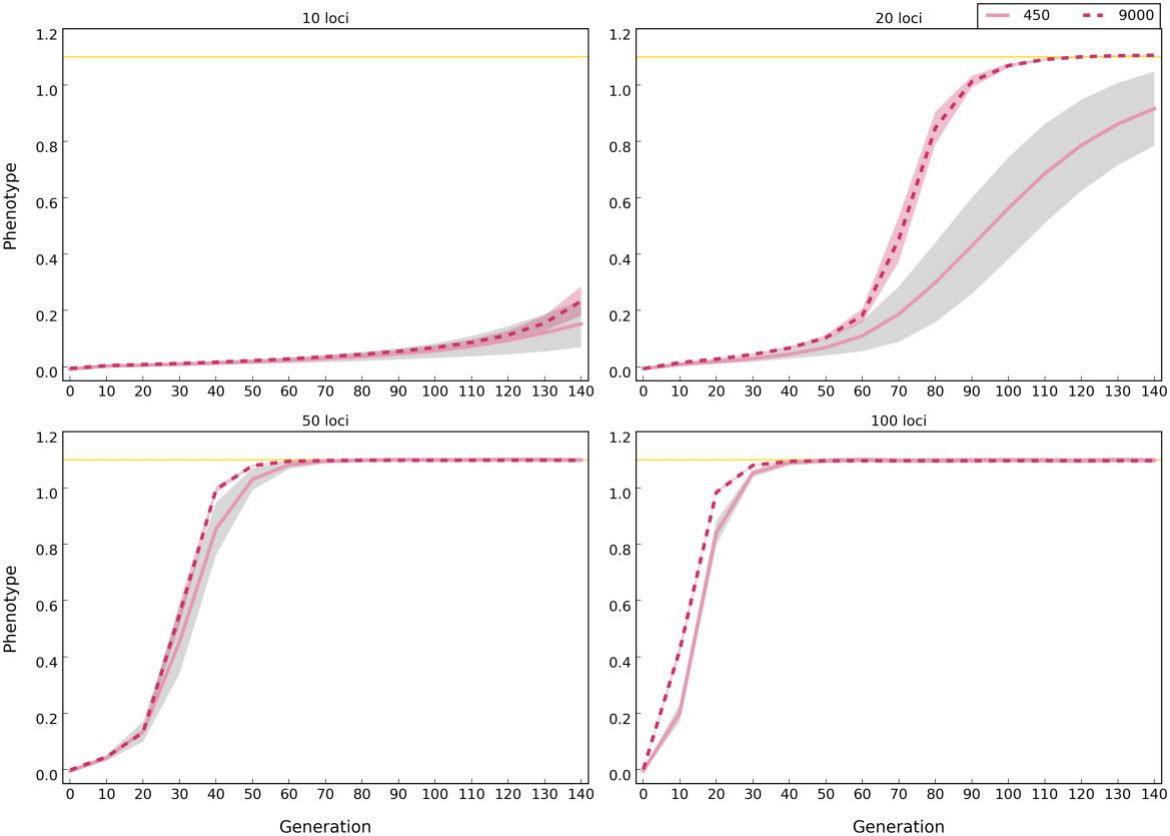
—Populations of 450 (solid lines) and 9,000 (dotted lines) individuals reach the phenotypic optimum at different time points (scenario B in table 1*b*). Lines show the median phenotype of populations averaged across 500 replicates and the shaded area around lines show standard deviation. The optimum phenotype is 1.1 (shown by yellow lines). Note that the distance between the population phenotype at generation 0 and the phenotypic optimum is the same across simulations with different number of beneficial loci. The plotted phenotype is normalized to account for different phenotypic means in the founder population; the mean phenotype of each population at generation 0 is subtracted from the phenotype of every individual at each time point.

**Fig. 6.**
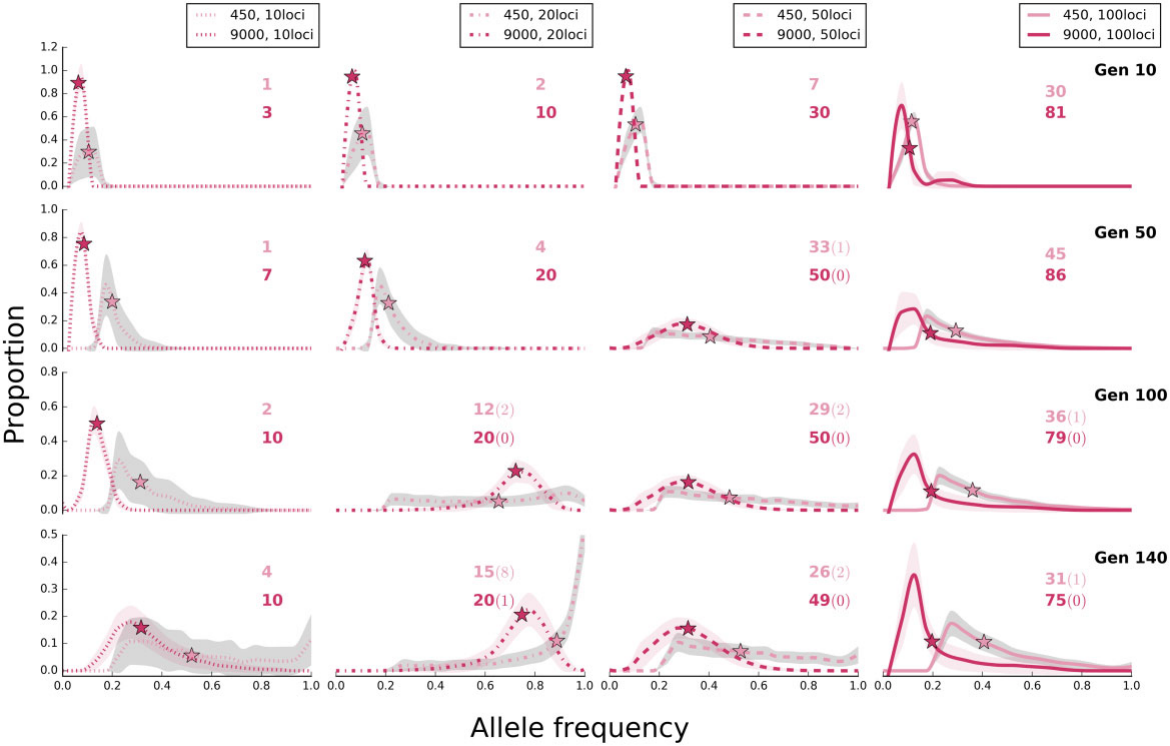
—Influence of population size and number of selection target on the site frequency spectrum of selected alleles for the trait optimum model. Populations with 450 and 9,000 individuals under trait optimum model are shown at different time points of the experiment (scenario B in table 1*b*). The lines (dotted: 10, dash dotted: 20, dashed: 50, and solid: 100 loci) show the average (binned from 0 to 1 with 0.05 intervals) frequency of selected alleles across 500 replicates and shaded areas depict standard deviation. On the *y* axis (proportion), we show the fraction of loci that experienced a larger frequency increase than expected under neutrality. Asterisks depict the median frequency increase of selected alleles averaged across 500 replicates. The number of alleles with frequency increase averaged across 500 replicates is shown with colors corresponding to the labels. The number of alleles with sweep-like signature (frequency ≥ 0.9) averaged across 500 replicates, if present, is shown in parentheses. Rows correspond to time points of the experiments, that is, generation, and shown as “Gen #.” The site frequency spectra of selected alleles in ten generation intervals are presented in supplementary figure S7, Supplementary Material online.

In the sweep model, fitness of populations with more selected alleles is greater than that of populations with fewer alleles (supplementary fig. S5, Supplementary Material online) due to the frequency increase of more selected alleles throughout the time (supplementary fig. S6, Supplementary Material online). For the trait optimum, we noticed a marked difference for founder populations with few alleles (e.g., 10 and 20), as in these simulations the trait optimum could not be reached (fig. 5, see supplementary fig. S7, Supplementary Material online, for site frequency spectra across 140 generations in ten-generation intervals), hence, no genetic redundancy was observed. In populations without redundancy (e.g., with 10 and 20 loci) and the sweep model, the median allele frequency continues to increase such that the two models cannot be distinguished (fig. 6).

Nevertheless, we noticed an interesting pattern: Under the sweep model in small populations, the fraction of selected alleles (loci with frequency change more than expected under drift) decreases as the number of beneficial loci in the founder population increases (supplementary fig. S6, Supplementary Material online). Barton (1995) showed that selection at linked loci causes variation in fitness and reduces the probability of fixation of selected loci. In our simulations, for populations with more selected loci (e.g., 100), recombination generates haplotypes with large variance in the number of selected alleles (supplementary fig. S8, Supplementary Material online, top panel). Variance in the number of selected alleles in turn increases the variance in fitness for those populations (supplementary fig. S8, Supplementary Material online, bottom panel), ultimately leading to the loss of some selected alleles by genetic drift. As a consequence, under sweep model, the similarity among replicates in populations with fewer selected alleles is greater than those with more alleles (supplementary fig. S9*A*, Supplementary Material online).

### Importance of Allelic Effect Size

**Fig. 7.**
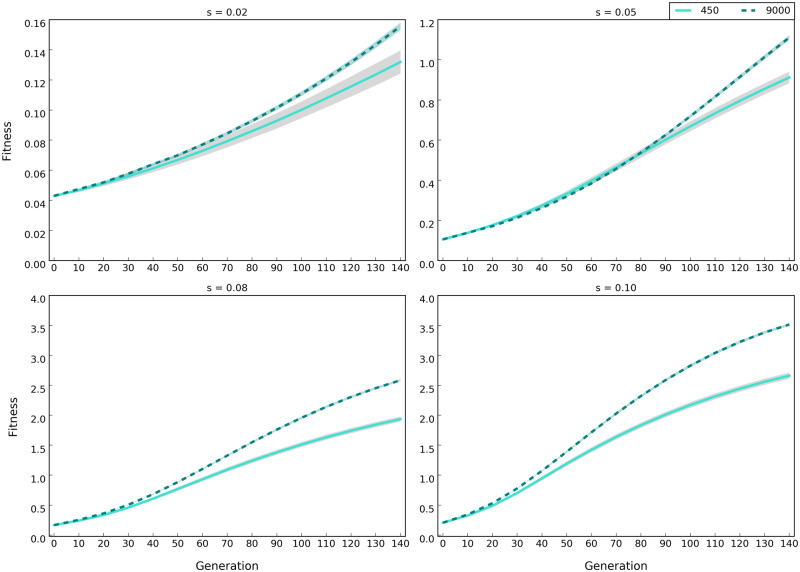
—Larger effect sizes result in higher fitness under the sweep model. Lines show the median fitness of the populations with 450 (solid lines) and 9,000 (dotted lines) individuals (averaged across 500 replicates) with 100 alleles of different selection coefficients (0.02, 0.05, 0.08, and 0.1) (scenario C in table 1*a*) and the shaded area show standard deviation. Fitness is log_10_ transformed.

**Fig. 8.**
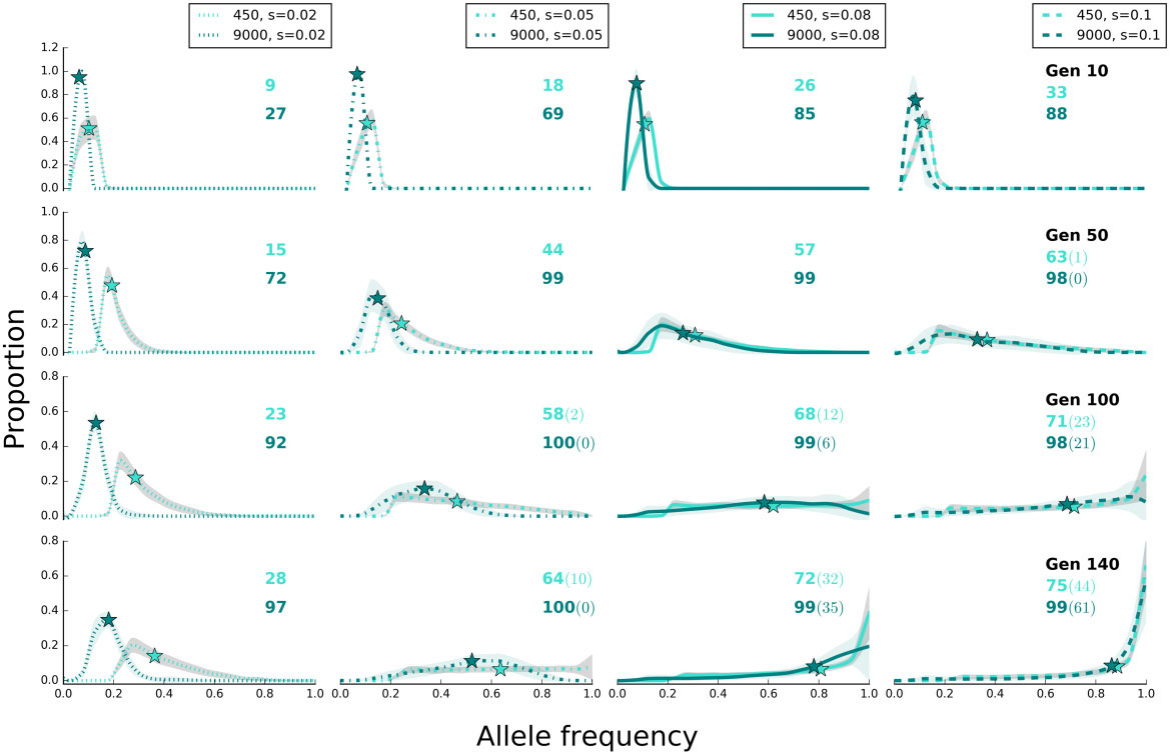
—Effect size determines the site frequency spectrum under sweep model. Populations with 450 and 9,000 individuals have a characteristic site frequency spectrum of selected alleles at different time points of the experiment (scenario C in table 1*b*). The lines (dotted: *s *=* *0.02, dash dotted: *s *=* *0.05, solid: *s *=* *0.08, and dashed: *s *=* *0.1) show the average (binned from 0 to 1 with 0.05 intervals) frequency of selected alleles across 500 replicates and shaded areas depict standard deviation. On the *y* axis (proportion), we show the fraction of loci that experienced a larger frequency increase than expected under neutrality. Asterisks depict the median frequency increase of selected alleles averaged across 500 replicates. The number of alleles with frequency increase averaged across 500 replicates is shown with colors corresponding to the labels. The number of alleles with sweep-like signature (frequency ≥ 0.9) averaged across 500 replicates, if present, is shown in parentheses. Rows correspond to time points of the experiments, that is, generation, and shown as “Gen #.” The site frequency spectra of selected alleles in ten generation intervals are presented in supplementary figure S10, Supplementary Material online.

**Fig. 9.**
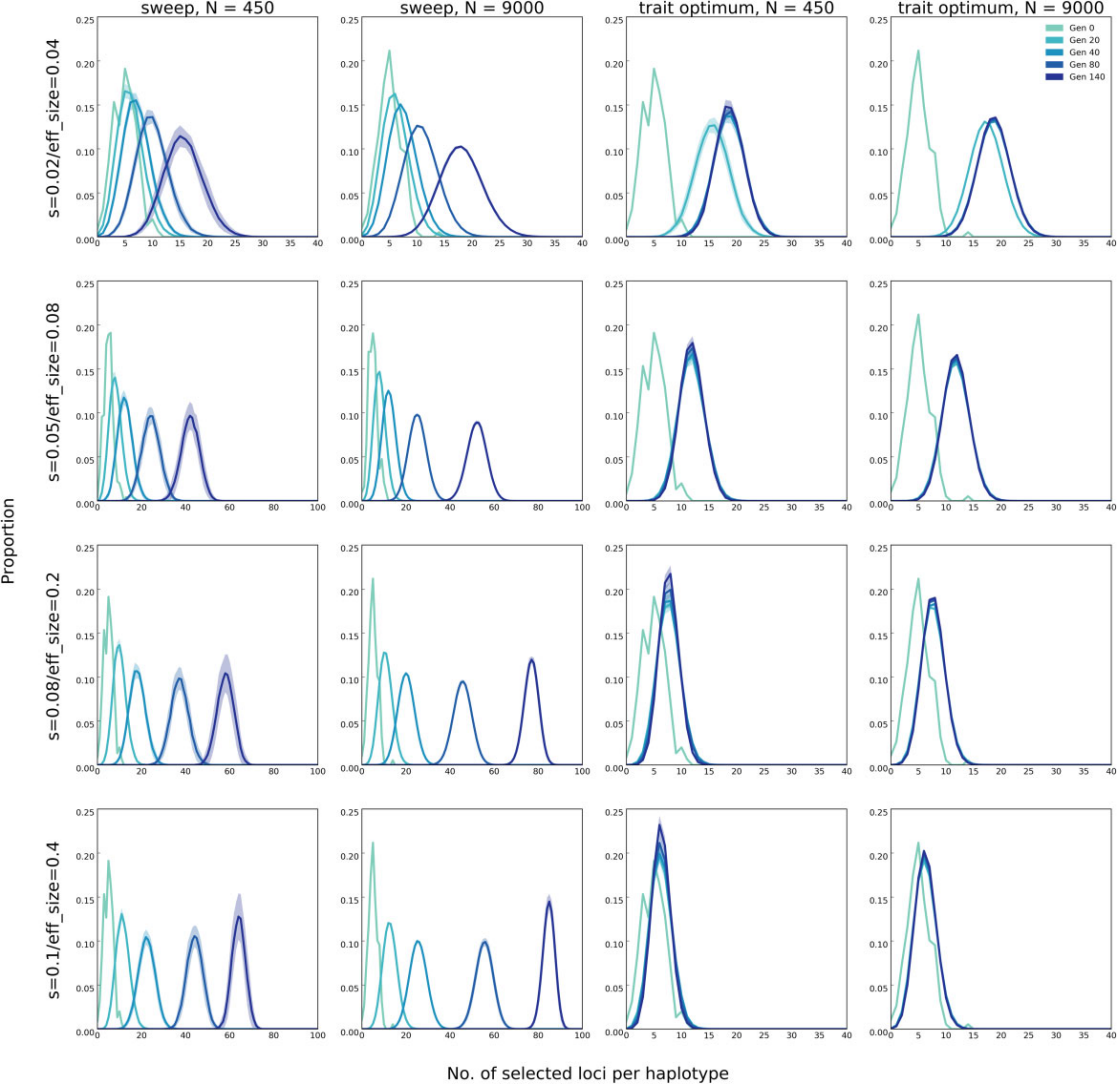
—Distribution of the number of beneficial loci per haplotype is affected by the effect size of loci under sweep and trait optimum models in populations of 450 and 9,000 individuals (scenario C in table 1*a* and *b*). Lines show the number of beneficial loci per haplotype (binned from 0 to 100 with intervals of 1) averaged across 50 replicates and the shaded area around lines show standard deviation. On the *y* axis (proportion), we show the fraction of haplotypes with specific number of beneficial loci from the total number of haplotypes in each replicate. Time points, that is, generation, are shown as “Gen #.” *s*, selection coefficient; eff_size, effect size.

We evaluated the influence of allelic effect size by simulating 100 linked loci with starting frequency of 0.05 and varying effect sizes (scenario C in table 1*a*, for sweep, and table 1*b*, for trait optimum model) in small and large populations in 500 iterations. Effect size has pronounced influence on the evolutionary trajectories. The fitness gain is higher and faster when alleles have larger effect sizes, that is, higher selection coefficients, under sweep model (fig. 7). Sweep signatures also become more frequent (fig. 8). As the effect size of beneficial alleles increases, the rise in frequency is higher (fig. 8 see supplementary fig. S10, Supplementary Material online, for site frequency spectra across 140 generations in ten-generation intervals), resulting in more beneficial loci recombining on the same haplotypes (fig. 9). This results in a higher parallelism among replicates (supplementary fig. S9B, Supplementary Material online). Under the trait optimum model, the trait optimum is also reached faster with alleles of larger effect sizes (supplementary fig. S11, Supplementary Material online) because smaller frequency shifts are required to achieve the same phenotypic change as the alleles of small effect size (supplementary fig. S12, Supplementary Material online). Thus, the signatures of reaching trait optimum are seen at earlier time points; drift reduces parallelism among replicates so that similarity among replicates, measured by Jaccard index, is almost as much as neutral alleles (trait optimum with effect size 0.2 and 0.4 in supplementary fig. S9*B*, Supplementary Material online), and the number of beneficial alleles per haplotypes remains stable (trait optimum with effect size 0.2 and 0.4 in fig. 9). Except for minor differences, the main distinctions between sweep and trait optimum models are not affected by allelic effect sizes.

One example for qualitatively different signatures can be found for the sweep model where the median frequency of the selected alleles with small selection coefficient (*s *=* *0.02) continues to increase faster in small populations than the subtle changes in the large ones (fig. 8) because drift acts synergistically for some alleles that are at a low frequency. A similar pattern can be seen for the trait optimum model (supplementary fig. S12, Supplementary Material online). Nevertheless, for the sweep model, the number of identified selection targets, even when *s* is small, increases as populations evolve (fig. 8), whereas due to drift it decreases for the trait optimum model (supplementary fig. S12, Supplementary Material online). These distinctive patterns can be used for differentiating the models.

## Conclusions

Our computer simulations identified several features that can be used to distinguish between the selective sweep and trait optimum models. Although one distinguishing feature requires phenotypic data, majority of features can be inferred from genomic data alone provided that reaching the trait optimum is assured.

1. The fitness of large populations is greater than that of small ones under the sweep model and continues to increase until the fixation of all selected alleles (fig. 2). For the trait optimum model, however, the fitness between small and large populations differs only until the trait optimum is reached and is not affected by further AFCs (fig. 2). No genetic data are required for this distinguishing feature.
2. The selected alleles increase in frequency until fixation under the sweep model, whereas the frequency of selected alleles increases until the phenotypic optimum is reached in the trait optimum model (fig. 1). After reaching the trait optimum, the median allele frequencies plateau in large populations but not in small populations. Strong drift and frequency decrease of selected alleles below detection limit are responsible for continued increase of the median allele frequencies. Nevertheless, the number of identified selected alleles is strongly reduced in later generations in small populations (fig. 1). Therefore, there is a clear difference between the two models in either small or large populations provided that the experiment is conducted for a sufficient number of generations.
3. The number of selected alleles shared among replicates (parallelism) is another distinguishing feature between the models. The parallelism among replicates continues to increase in the sweep model, whereas after reaching the trait optimum repeatability of adaptation decreases under the trait optimum model (fig. 3). Therefore, replication provides a powerful means for distinguishing evolutionary models.
4. The number of beneficial alleles per haplotype continues to increase in the sweep model, whereas after reaching the phenotypic optimum, it plateaus under the trait optimum model (fig. 4). This feature requires availability of phased haplotypes but provides another confirmatory test for distinguishing the two evolutionary models.

Despite the presence of several distinct features to differentiate the two models, discerning the models is limited under specific conditions. As a consequence of consistent frequency increase in the sweep model, many alleles reach frequencies close to fixation, that is, sweep-like signatures (fig. 1). However, sweep-like signatures were extremely rare for the trait optimum model (fig. 1). Nevertheless, we caution that this is not a very reliable discriminating feature. Under sweep model, if the effect size of contributing loci is small AFC is very small and fixation is not seen because the duration of the experiments is not sufficiently long. On the other hand, if the genetic diversity in the founder population is low, that is, few beneficial loci (fig. 6), or the new trait optimum is far from the median phenotypic state of the founder population allele frequencies will have sweep-like signatures under the trait optimum model. Frequency change of alleles with small effect size under sweep model is so small (allele frequency trajectories for *s *=* *0.02 in fig. 8) that they may become fixed after a long time. In this case, the pattern of AFCs during the course of experiment resembles the plateau in allele frequencies observed in trait optimum model (e.g., in fig. 1), and allele frequencies cannot serve as a discriminating feature. Unlike trait optimum model, the number of selected alleles increases throughout the experiment (fig. 8). In addition, other distinctive features such as the consistent increase in fitness, high parallelism among replicates, and haplotype structure will have sweep-specific features.

The main characteristics to distinguish the two models are the temporal changes in allele frequencies, phenotype and haplotype structure which require availability of time series data (Pool-Seq for accurate estimation of allele frequencies in populations and haplotype sequences for inference of linkage disequilibrium, LD, among selected sites). Experimental evolution provides the opportunity for collecting time series data but availability of such data may not be possible for most natural systems. Fossils can be used but they are rarely available, and if present, can only provide information about allele frequencies. Sampling populations from the same location in consecutive years can be a useful approach (Bergland et al. 2014). However, factors such as migration cannot be ruled out. Also, the selection signature signals might be confounded by other unknown environmental changes.

Another key feature to distinguish sweep and trait optimum models is the level of parallelism among replicates. Availability of replication is generally not a constraint in experimental evolution studies but can only be obtained from specific natural study systems. It should be noted that natural populations that can be considered as replicates are generally exposed to similar, but not identical, environmental conditions. Moreover, local adaptation can reduce the similarity among replicates (Stuart et al. 2017). Several natural study systems are available for investigating polygenic adaptation although the level of replication varies among them. For example, latitudinal (Adrion et al. 2015) and altitudinal (Bigham et al. 2010; Yi et al. 2010; Keller et al. 2013; Crawford et al. 2017; Fior et al. 2018) clines where populations are exposed to similar conditions, populations of cichlids that have independently colonized lakes (Elmer and Meyer 2011; Meier et al. 2018), guppy populations in rivers of the Northern Mountain range in Trinidad that are exposed to high and low predation (Alexander et al. 2006; Hendry et al. 2006), and probably the system with highest level of replication are sticklebacks in multiple rivers that allow contrasts of marine and freshwater, lake and stream, and benthic and limnetic habitats (Hohenlohe et al. 2010; Jones, Chan, et al. 2012; Jones, Grabherr, et al. 2012).

A variety of methods have been developed for the identification of selective sweeps. These methods rely on specific features of the genomic region under selection including reduced variation, change in patterns of LD, or shift in site frequency spectrum (e.g., Messer and Petrov 2013; Pavlidis and Alachiotis 2017). Several tests for identification of polygenic adaptation have also been developed. Selected variants are either identified via the correlation of single-nucleotide polymorphism (SNP) frequencies in multiple populations with environmental variables (Coop et al. 2010) or using Gene Ontology categories or pathways (Daub et al. 2013; Gouy et al. 2017). However, these methods do not provide information about the causal relationship between the selected variants and phenotypes. Availability of phenotypes and genotypes in genome-wide association studies allows identification of a group of SNPs with significant association (statistically different from the background SNPs) with the focal trait (Turchin et al. 2012). This approach has been used for identification of polygenic basis for waist-to-hip ratio and height in human populations (Field et al. 2016). Alternatively, not only the significant SNPs but all SNPs weighted according to the explained phenotypic variance are used (Berg and Coop 2014). Using this approach, selection signatures for traits including body size and skin pigmentation in human populations are identified (Novembre and Barton 2018). Here, we identified several discriminating features for sweep and trait optimum models and showed that the combination of large and small replicate populations further uncovers some distinctive patterns that can be used for developing test statistics to discriminate between the two models. Machine learning has been used for identification of loci under hard sweep, soft sweep, and balancing selection (Lin et al. 2011; Schrider and Kern 2016, 2018; Sheehan and Song 2016; Kern and Schrider 2018). We propose that machine learning could be a powerful approach to also exploit the described features for a quantitative approach to distinguish between the two models. We consider combining the analysis of small and large populations as a suitable means for the analysis of the adaptive architectures. Large populations clearly offer the advantage to identify a larger number of selected alleles which increase in frequency in multiple replicates. Small populations are easier and cheaper to maintain while still offering discriminative features. However, mapping the causative variant will be more challenging in small populations because of stronger LD and more confounding signal from neutral alleles.

## Data Availability

Scripts to generate files needed for simulations, command lines for simulations, and scripts for data analysis and visualization are provided in https://github.com/popgenvienna/SweepVersusTraitOptimum (last accessed April 20, 2020).

## Supplementary Material

evaa073_Supplementary_Data

## References

[evaa073-B1] Adrion JR, Hahn MW, Cooper BS. 2015 Revisiting classic clines in *Drosophila melanogaster* in the age of genomics. Trends Genet. 31(8):434–444. 26072452 10.1016/j.tig.2015.05.006 PMC4526433

[evaa073-B2] Alexander AHJ, Taylor JS, Wu SS, Breden F. 2006 Parallel evolution and vicariance in the guppy (*Poecilia reticulata*) over multiple spatial and temporal scales. Evolution 60(11):2352–2369. 17236426

[evaa073-B3] Barghi N, et al 2019 Genetic redundancy fuels polygenic adaptation in *Drosophila*. PLoS Biol. 17(2):e3000128. 30716062 10.1371/journal.pbio.3000128 PMC6375663

[evaa073-B4] Barton NH. 1995 Linkage and the limits to natural selection. Genetics 140(2):821–841. 7498757 10.1093/genetics/140.2.821 PMC1206655

[evaa073-B5] Barton NH, Etheridge AM, Véber A. 2017 The infinitesimal model: definition, derivation, and implications. Theor Popul Biol. 118:50–73. 28709925 10.1016/j.tpb.2017.06.001

[evaa073-B6] Berg JJ, Coop G. 2014 A population genetic signal of polygenic adaptation. PLoS Genet. 10(8):e1004412. 25102153 10.1371/journal.pgen.1004412 PMC4125079

[evaa073-B7] Bergland AO, Behrman EL, O’Brien KR, Schmidt PS, Petrov DA. 2014 Genomic evidence of rapid and stable adaptive oscillations over seasonal time scales in *Drosophila*. PLoS Genet. 10(11):e1004775. 25375361 10.1371/journal.pgen.1004775 PMC4222749

[evaa073-B8] Bigham A, et al 2010 Identifying signatures of natural selection in Tibetan and Andean populations using dense genome scan data. PLoS Genet. 6(9):e1001116. 20838600 10.1371/journal.pgen.1001116 PMC2936536

[evaa073-B9] Burke MK, et al 2010 Genome-wide analysis of a long-term evolution experiment with *Drosophila*. Nature 467(7315):587–590. 20844486 10.1038/nature09352

[evaa073-B10] Chevin LM, Hospital F. 2008 Selective sweep at a quantitative trait locus in the presence of background genetic variation. Genetics 180(3):1645–1660. 18832353 10.1534/genetics.108.093351 PMC2581964

[evaa073-B11] Conte GL, et al 2015 Extent of QTL reuse during repeated phenotypic divergence of sympatric threespine stickleback. Genetics 201(3):1189–1200. 26384359 10.1534/genetics.115.182550 PMC4649644

[evaa073-B12] Coop G, Witonsky D, Di Rienzo A, Pritchard JK. 2010 Using environmental correlations to identify loci underlying local adaptation. Genetics 185(4):1411–1423. 20516501 10.1534/genetics.110.114819 PMC2927766

[evaa073-B13] Crawford JE, et al 2017 Natural selection on genes related to cardiovascular health in high-altitude adapted Andeans. Am J Hum Genet. 101(5):752–767. 29100088 10.1016/j.ajhg.2017.09.023 PMC5673686

[evaa073-B14] Daub JT, et al 2013 Evidence for polygenic adaptation to pathogens in the human genome. Mol Biol Evol. 30(7):1544–1558. 23625889 10.1093/molbev/mst080

[evaa073-B15] Elmer KR, Meyer A. 2011 Adaptation in the age of ecological genomics : insights from parallelism and convergence. Trends Ecol Evol. 26(6):298–306. 21459472 10.1016/j.tree.2011.02.008

[evaa073-B16] Field Y, et al 2016 Detection of human adaptation during the past 2,000 years. Science 354(6313):760–764. 27738015 10.1126/science.aag0776 PMC5182071

[evaa073-B17] Fior S, et al 2018 Trait differentiation and adaptation of plants along elevation gradients. J Evol Biol. 31(6):784–800. 29518274 10.1111/jeb.13262

[evaa073-B18] Franssen SU, Kofler R, Schlötterer C. 2017 Uncovering the genetic signature of quantitative trait evolution with replicated time series data. Heredity (Edinb). 118(1):42–51. 27848948 10.1038/hdy.2016.98 PMC5176121

[evaa073-B19] Goldstein DB, Holsinger KE. 1992 Maintenance of polygenic variation in spacially structured populations: roles for local mating and genetic redundancy. Evolution 46(2):412–429. 28564040 10.1111/j.1558-5646.1992.tb02048.x

[evaa073-B20] Gouy A, Daub T, Excoffier L. 2017 Detecting gene subnetworks under selection in biological pathways. Nucleic Acids Res. 45(16):e149. 28934485 10.1093/nar/gkx626 PMC5766194

[evaa073-B21] Hayward LK, Sella G. 2019. Polygenic adaptation after a sudden change in environment. bioRxiv 792952, doi: https://doi.org/10.1101/792952. 10.7554/eLife.66697 PMC9683794 36155653

[evaa073-B22] Hendry AP, Kelly ML, Kinnison MT, Reznick DN. 2006 Parallel evolution of the sexes? Effects of predation and habitat features on the size and shape of wild guppies. J Evol Biol. 19(3):741–754. 16674571 10.1111/j.1420-9101.2005.01061.x

[evaa073-B23] Hermisson J, Pennings PS. 2005 Soft sweeps: molecular population genetics of adaptation from standing genetic variation. Genetics 169(4):2335–2352. 15716498 10.1534/genetics.104.036947 PMC1449620

[evaa073-B24] Hohenlohe PA, et al 2010 Population genomics of parallel adaptation in threespine stickleback using sequenced RAD tags. PLoS Genet. 6(2):e1000862. 20195501 10.1371/journal.pgen.1000862 PMC2829049

[evaa073-B25] Höllinger I, Pennings P, Hermisson J. 2019 Polygenic adaptation : from sweeps to subtle frequency shifts. PLoS Genet. 15(3):e1008035. 30893299 10.1371/journal.pgen.1008035 PMC6443195

[evaa073-B26] Howie JM, Mazzucco R, Taus T, Nolte V, Schlötterer C. 2019 DNA motifs are not general predictors of recombination in two *Drosophila* sister species. Genome Biol Evol. 11(4):1345–1357. 30980655 10.1093/gbe/evz082 PMC6490297

[evaa073-B27] Jaccard P. 1901 Étude comparative de la distribution florale dans une portion des Alpes et des Jura. Bull Soc Vaudoise Sci Nat. 37 (142):547–579.

[evaa073-B28] Jain K, Stephan W. 2017 Rapid adaptation of a polygenic trait after a sudden environmental shift. Genetics 206(1):389–406. 28341654 10.1534/genetics.116.196972 PMC5419483

[evaa073-B29] Jones FC, Chan YF, et al 2012 A genome-wide SNP genotyping array reveals patterns of global and repeated species-pair divergence in sticklebacks. Curr Biol. 22(1):83–90. 22197244 10.1016/j.cub.2011.11.045 PMC3319444

[evaa073-B30] Jones FC, Grabherr MG, et al 2012 The genomic basis of adaptive evolution in threespine sticklebacks. Nature 484(7392):55–61. 22481358 10.1038/nature10944 PMC3322419

[evaa073-B31] Kawecki TJ, et al 2012 Experimental evolution. Trends Ecol Evol (Amst). 27(10):547–560. 22819306 10.1016/j.tree.2012.06.001

[evaa073-B32] Keller I, Alexander JM, Holderegger R, Edwards PJ. 2013 Widespread phenotypic and genetic divergence along altitudinal gradients in animals. J Evol Biol. 26(12):2527–2543. 24128377 10.1111/jeb.12255

[evaa073-B33] Kern AD, Schrider DR. 2018 diploS/HIC : an updated approach to classifying selective sweeps. G3 (Bethesda) 8(6):1959–1970. 29626082 10.1534/g3.118.200262 PMC5982824

[evaa073-B34] Lin K, Li H, Schlötterer C, Futschik A. 2011 Distinguishing positive selection from neutral evolution: boosting the performance of summary statistics. Genetics 187(1):229–244. 21041556 10.1534/genetics.110.122614 PMC3018323

[evaa073-B36] Maynard Smith J, Haigh J. 1974 The hitch-hiking effect of a favourable gene. Genet Res. 23(1):23–35. 4407212

[evaa073-B37] Meier JI, Marques DA, Wagner CE, Excoffier L, Seehausen O. 2018 Genomics of parallel ecological speciation in lake Victoria cichlids. Mol Biol Evol. 35(6):1489–1506. 29617828 10.1093/molbev/msy051

[evaa073-B38] Messer PW, Petrov DA. 2013 Population genomics of rapid adaptation by soft selective sweeps. Trends Ecol Evol (Amst). 28(11):659–669. 24075201 10.1016/j.tree.2013.08.003 PMC3834262

[evaa073-B39] Novembre J, Barton NH. 2018 Tread lightly interpreting polygenic tests of selection. Genetics 208(4):1351–1355. 29618592 10.1534/genetics.118.300786 PMC5886544

[evaa073-B40] Nowak MA, Boerlijst MC, Cooke J, Smith JM. 1997 Evolution of genetic redundancy. Nature 388(6638):167–171. 9217155 10.1038/40618

[evaa073-B41] Pavlidis P, Alachiotis N. 2017 A survey of methods and tools to detect recent and strong positive selection. J Biol Res (Thessalon). 24:7. 28405579 10.1186/s40709-017-0064-0 PMC5385031

[evaa073-B42] Pritchard JK, Di Rienzo A. 2010 Adaptation—not by sweeps alone. Nat Rev Genet. 11(10):665–667. 20838407 10.1038/nrg2880 PMC4652788

[evaa073-B43] Pritchard JK, Pickrell JK, Coop G. 2010 The genetics of human adaptation: hard sweeps, soft sweeps, and polygenic adaptation. Curr Biol. 20(4):R208–R215. 20178769 10.1016/j.cub.2009.11.055 PMC2994553

[evaa073-B44] Przeworski M, Coop G, Wall JD. 2005 The signature of positive selection on standing genetic variation. Evolution 59(11):2312–2323. 16396172

[evaa073-B45] Schlötterer C, Kofler R, Versace E, Tobler R, Franssen SU. 2015 Combining experimental evolution with next-generation sequencing: a powerful tool to study adaptation from standing genetic variation. Heredity 114(5):431–440. 25269380 10.1038/hdy.2014.86 PMC4815507

[evaa073-B46] Schlötterer C, Tobler R, Kofler R, Nolte V. 2014 Sequencing pools of individuals—mining genome-wide polymorphism data without big funding. Nat Rev Genet. 15(11):749–763. 25246196 10.1038/nrg3803

[evaa073-B47] Schrider DR, Kern AD. 2016 S/HIC: robust identification of soft and hard sweeps using machine learning. PLoS Genet. 12(3):e1005928. 26977894 10.1371/journal.pgen.1005928 PMC4792382

[evaa073-B48] Schrider DR, Kern AD. 2018 Supervised machine learning for population genetics : a new paradigm. Trends Genet. 34(4):301–312. 29331490 10.1016/j.tig.2017.12.005 PMC5905713

[evaa073-B49] Sheehan S, Song YS. 2016 Deep learning for population genetic inference. PLoS Comput Biol. 12(3):e1004845. 27018908 10.1371/journal.pcbi.1004845 PMC4809617

[evaa073-B50] Stuart YE, et al 2017 Contrasting effects of environment and genetics generate a continuum of parallel evolution. Nat Ecol Evol. 1(6):1–7. 28812631 10.1038/s41559-017-0158

[evaa073-B51] Therkildsen NO, et al 2019 Contrasting genomic shifts underlie parallel phenotypic evolution in response to fishing. Science 365(6452):487–490. 31371613 10.1126/science.aaw7271

[evaa073-B52] Thornton KR. 2019 Polygenic adaptation to an environmental shift: temporal dynamics of variation under Gaussian stabilizing selection and additive effects on a single trait. Genetics 213(4):1513–1530. 31653678 10.1534/genetics.119.302662 PMC6893385

[evaa073-B53] Tobler R, et al 2014 Massive habitat-specific genomic response in *D. melanogaster* populations during experimental evolution in hot and cold environments. Mol Biol Evol. 31(2):364–375. 24150039 10.1093/molbev/mst205 PMC3907058

[evaa073-B54] Turchin MC, et al 2012 Evidence of widespread selection on standing variation in Europe at height-associated SNPs. Nat Genet. 44(9):1015–1019. 22902787 10.1038/ng.2368 PMC3480734

[evaa073-B55] Turner TL, Stewart AD, Fields AT, Rice WR, Tarone AM. 2011 Population-based resequencing of experimentally evolved populations reveals the genetic basis of body size variation in *Drosophila melanogaster*. PLoS Genet. 7(3):e1001336. 21437274 10.1371/journal.pgen.1001336 PMC3060078

[evaa073-B56] Vlachos C, Kofler R. 2018 MimicrEE2: genome-wide forward simulations of evolve and resequencing studies. PLoS Comput Biol. 14(8):e1006413–10. 30114186 10.1371/journal.pcbi.1006413 PMC6112681

[evaa073-B57] Yeaman S. 2015 Local adaptation by alleles of small effect. Am Nat. 186(S1):S74–S89. 26656219 10.1086/682405

[evaa073-B58] Yi X, et al 2010 Sequencing of 50 human exomes reveals adaptation to high altitude. Science 329(5987):75–78. 20595611 10.1126/science.1190371 PMC3711608

