# Supplementary material for: Distinct Patterns of Selective Sweep and Polygenic Adaptation in Evolve and Resequence Studies
Source: Genome Biol Evol. 2020 Apr 18;12(6):890–904. doi: 10.1093/gbe/evaa073 (PMC7313669; doi:10.1093/gbe/evaa073)
Supplement: evaa073_Supplementary_Data [file evaa073_supplementary_data.docx]

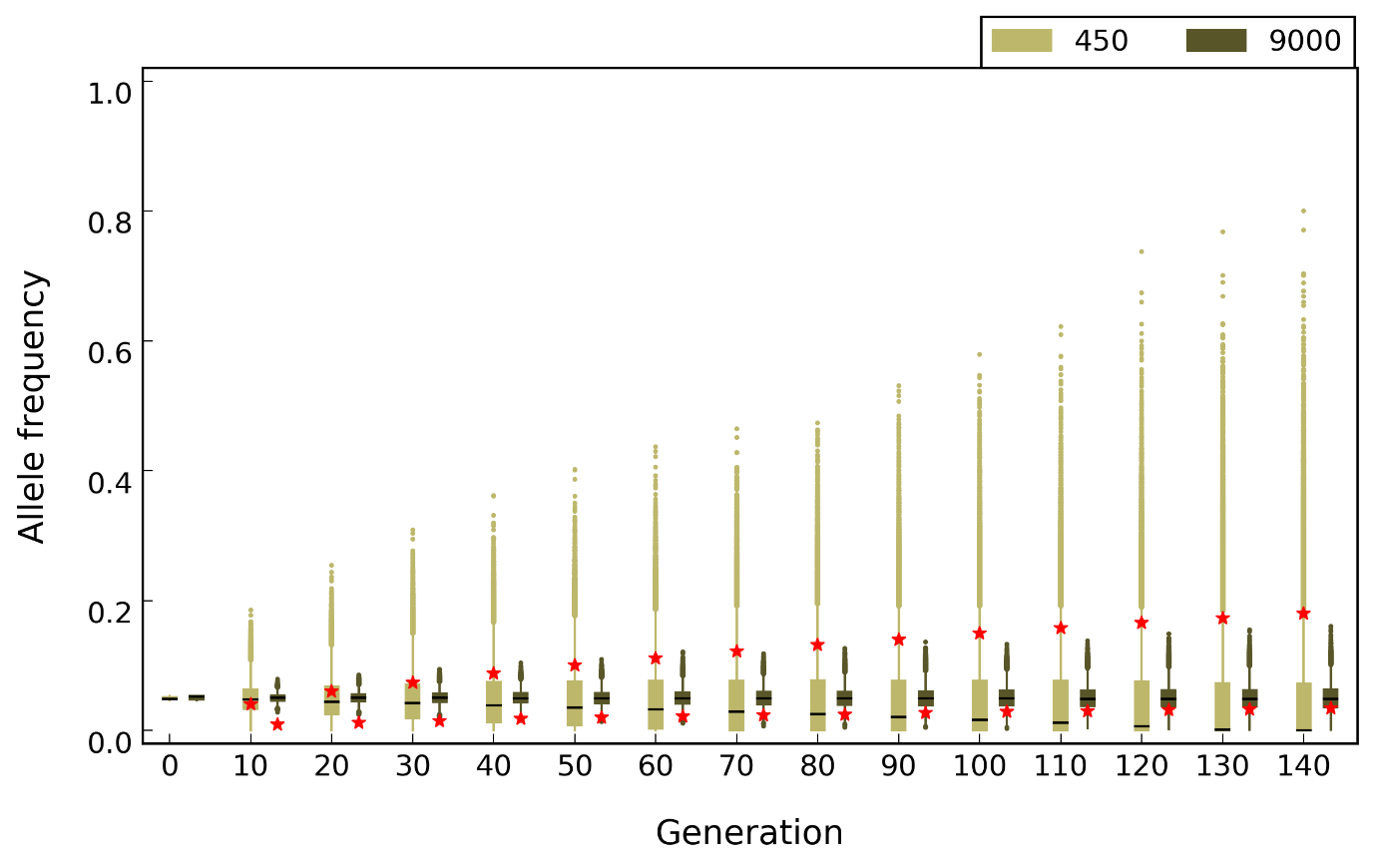


**Figure S1** Change in allele frequencies due to genetic drift in small populations (450 individuals) is higher than in large ones (9,000 individuals). Box plots show frequencies of 100 alleles under drift in 500 replicates (scenario A in Table 1b with no selection). Red asterisks depict the frequency cut-off based on 95% quantile of allele frequency change under neutral simulations.

**
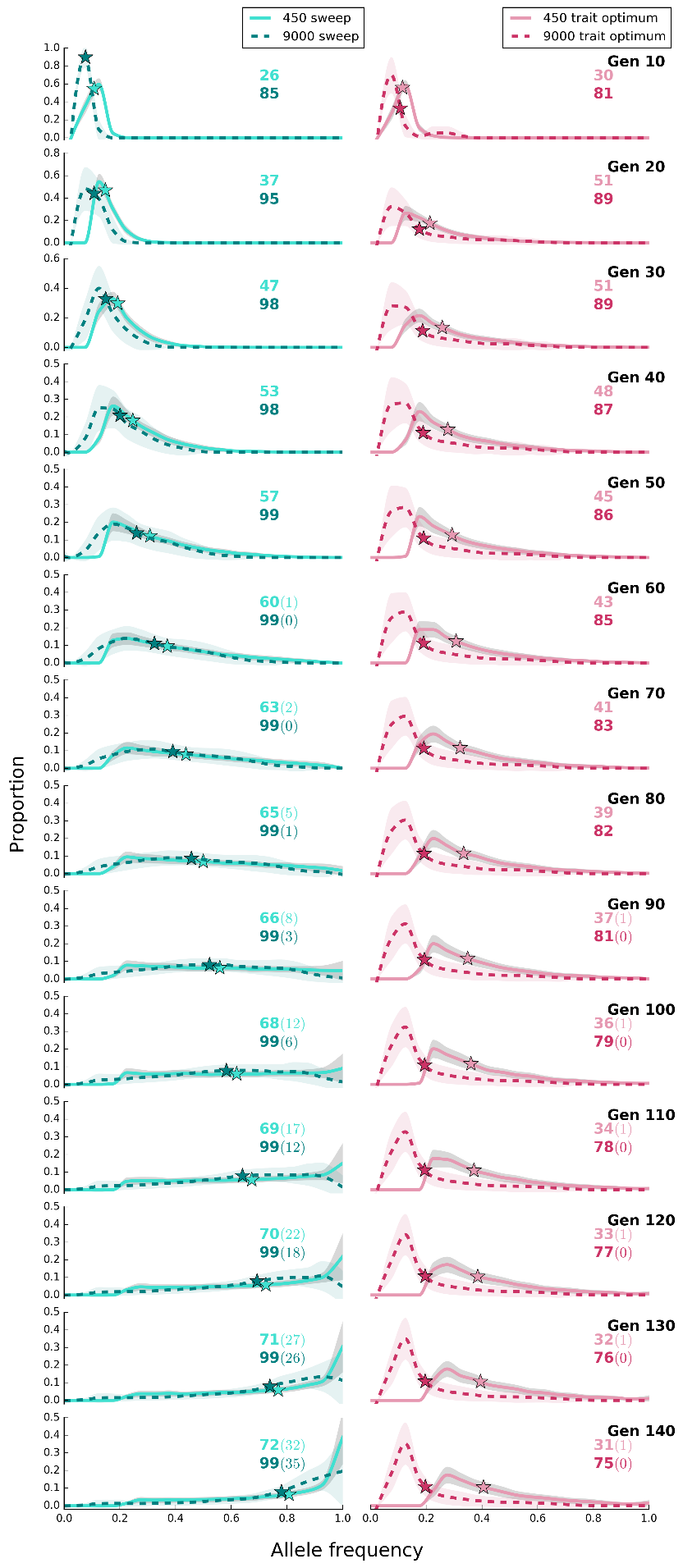
**

**Figure S2** The site frequency spectrum of selected alleles differs in large and small populations for the sweep and trait optimum models. Populations with 450 and 9,000 individuals evolved for 140 generations under the two different selection regimes (scenario A in Table 1a and 1b). The lines show the average (binned from 0 to 1 with 0.05 intervals) frequency of selected alleles across 500 replicates and shaded areas depict standard deviation. On the Y-axis (proportion) we show the fraction of loci that experienced a larger frequency increase than expected under neutrality. Asterisks depict the median frequency increase of selected alleles averaged across 500 replicates. The number of alleles with frequency increase averaged across 500 replicates is shown with colors corresponding to the labels. The number of alleles with sweep-like signature (frequency ≥ 0.9) averaged across 500 replicates, if present, is shown in parentheses. Rows correspond to time points of the experiments, i.e. generation, and shown as ‘Gen #’.

**
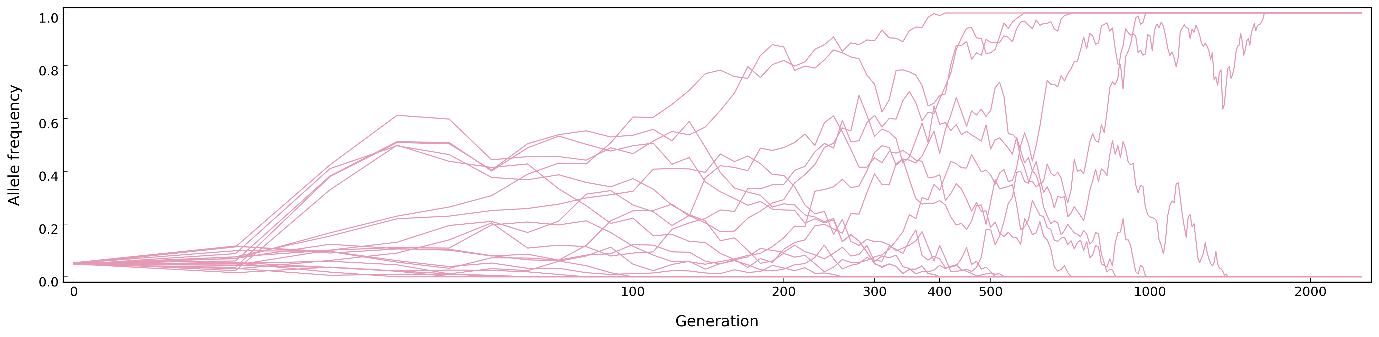
**

**Figure S3** Three phases of adaptation under trait optimum model. After reaching the trait optimum at generation 40 (phase 1), the drift phase (phase 2) starts. In phase 3, all alleles are sorted, i.e. either fixed or lost. Trajectories of selected alleles in a population of 450 individuals (scenario A in Table 1b) are shown. X axis is log10-transformed and for clarity only 20 out of 100 loci are shown.

**
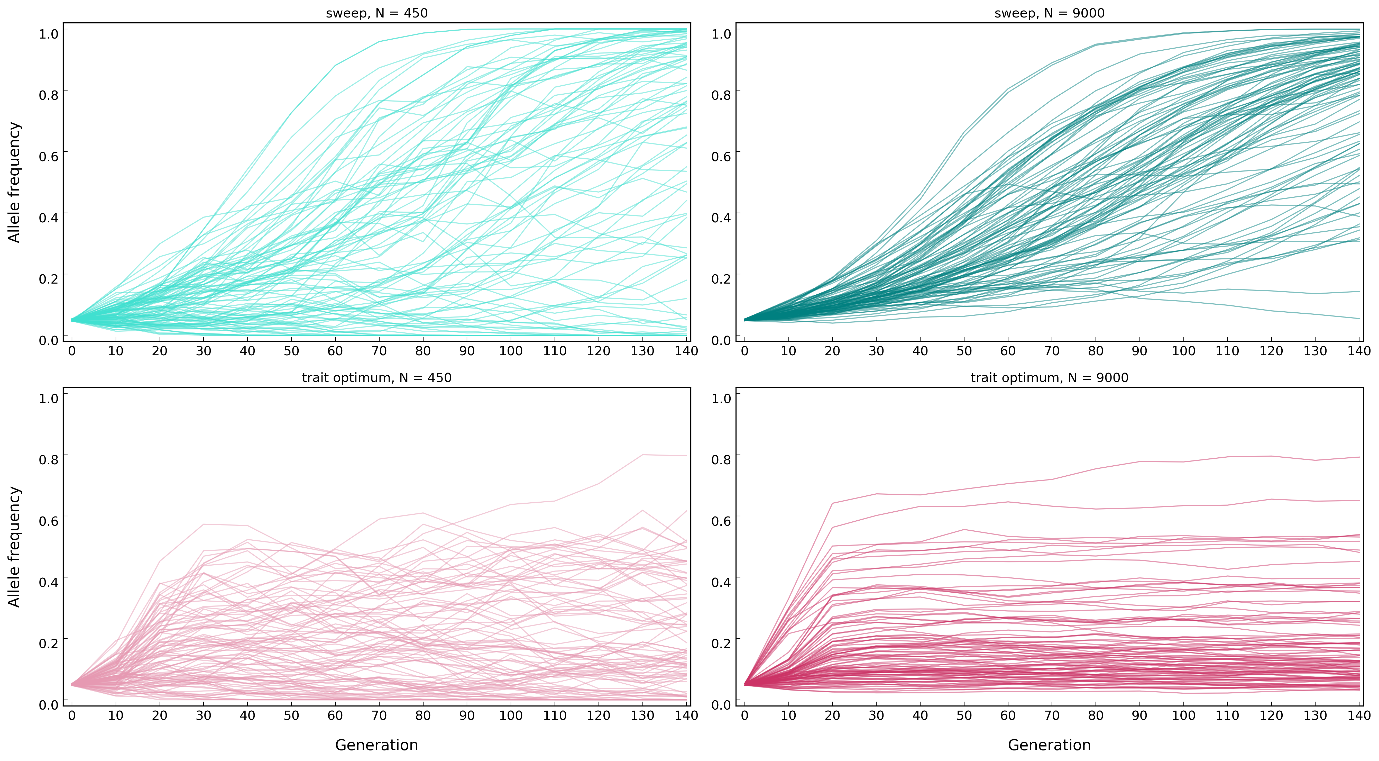
**

**Figure S4** Allele frequency trajectories of one replicate shown as an example for sweep (top panels) and trait optimum (bottom panels) models in populations of 450 and 9,000 individuals (scenario A in Table 1a and 1b).


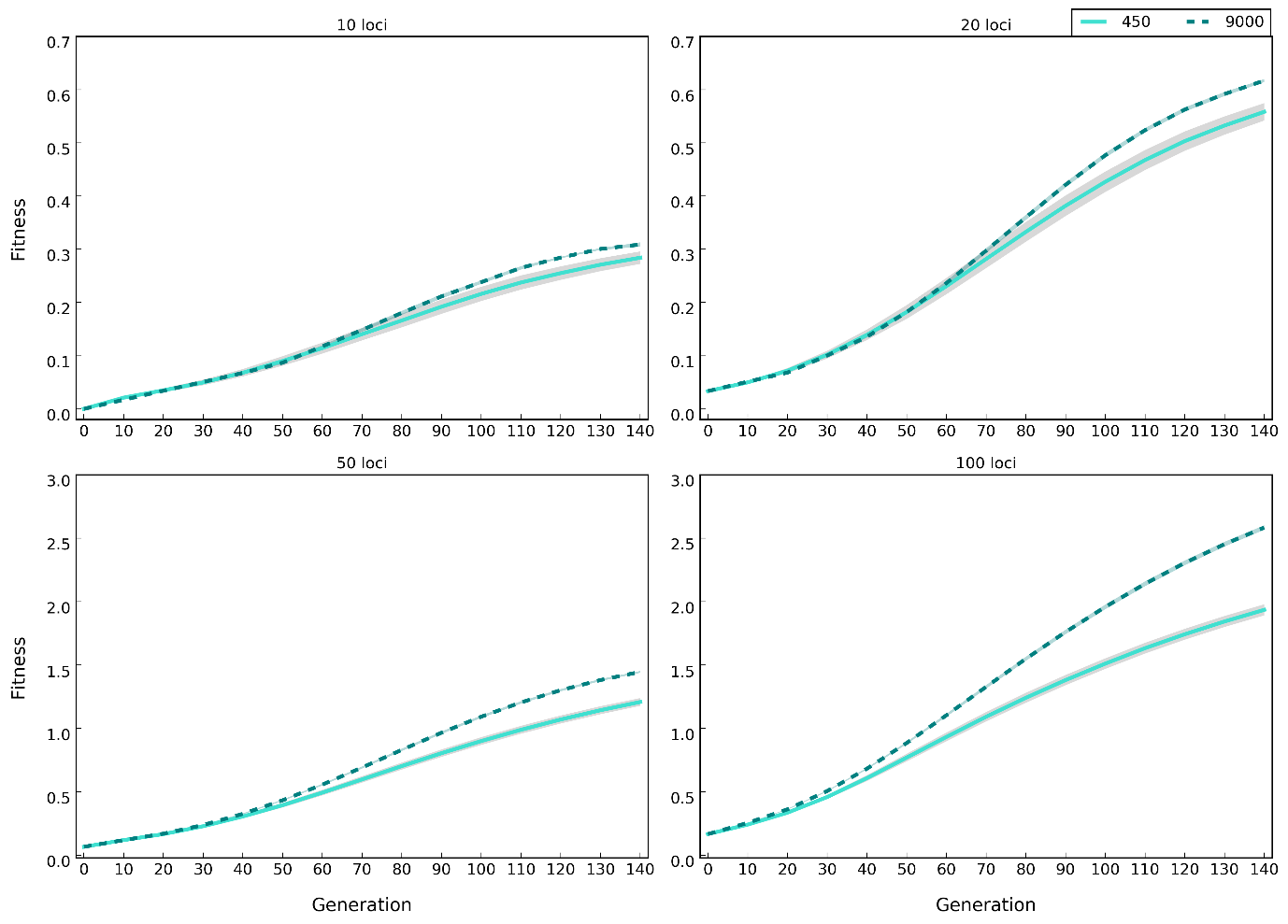


**Figure S5** Populations with more beneficial alleles have higher fitness under sweep model. Lines show the median fitness of populations with 450 (solid lines) and 9,000 (dotted lines) individuals (averaged across 500 replicates) with different number of beneficial loci (10, 20, 50, and 100) and the shaded area around lines show standard deviation (Scenario B in Table 1a). Fitness is log_10_ transformed.


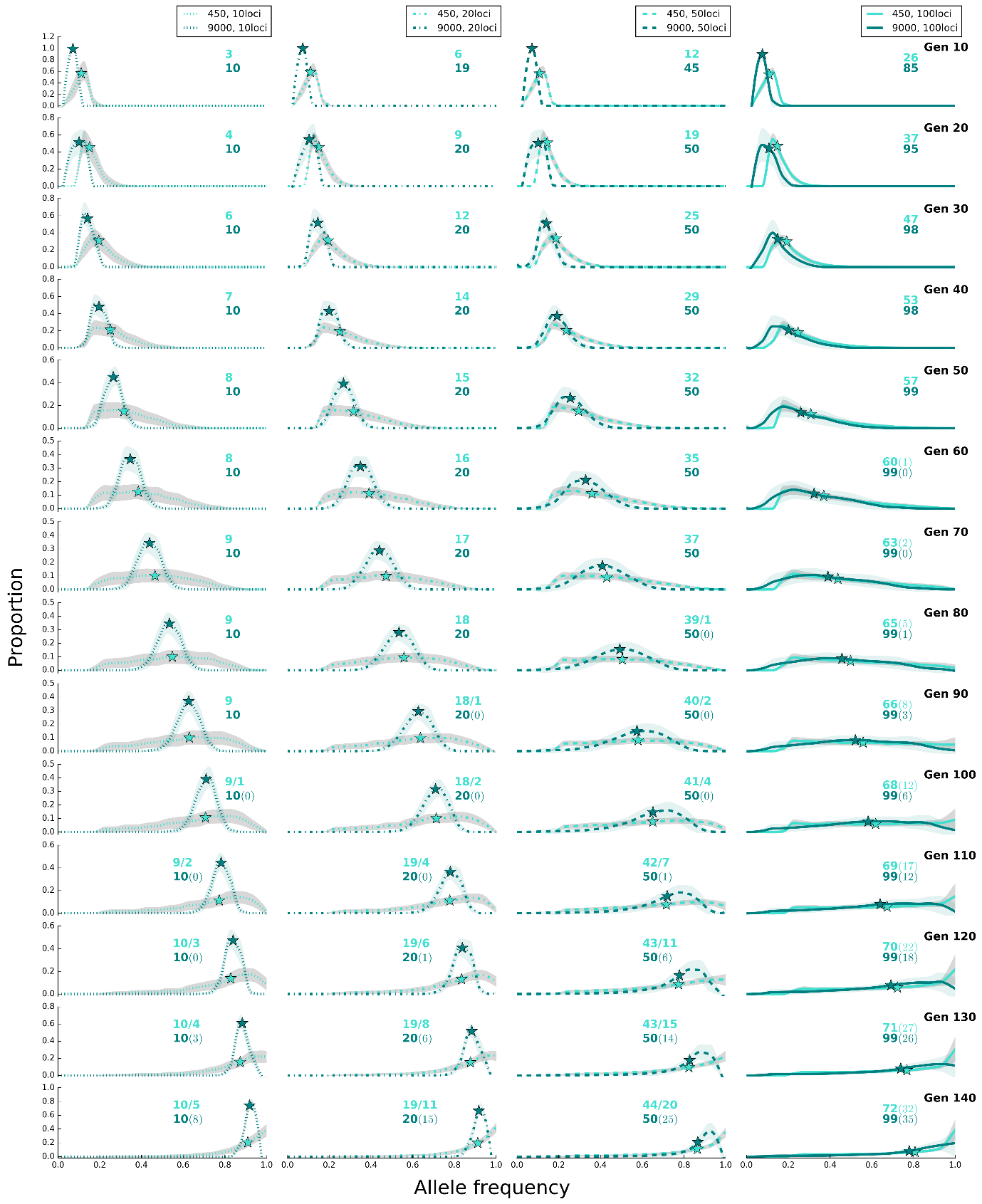


**Figure S6** Influence of population size and number of selection target on the site frequency spectrum of selected alleles for the sweep model. Populations with 450 and 9,000 individuals under sweep model are shown across 140 generations with 10-generation intervals (scenario B in Table 1a). The lines (dotted: 10, dash dotted: 20, dashed: 50, and solid: 100 loci) show the average (binned from 0 to 1 with 0.05 intervals) frequency of selected alleles across 500 replicates and shaded areas depict standard deviation. On the Y-axis (proportion) we show the fraction of loci that experienced a larger frequency increase than expected under neutrality. Asterisks depict the median frequency increase of selected alleles averaged across 500 replicates. The number of alleles with frequency increase averaged across 500 replicates is shown with colors corresponding to the labels. The number of alleles with sweep-like signature (frequency ≥ 0.9) averaged across 500 replicates, if present, is shown in parentheses. Rows correspond to time points of the experiments, i.e. generation, and shown as ‘Gen #’.

**
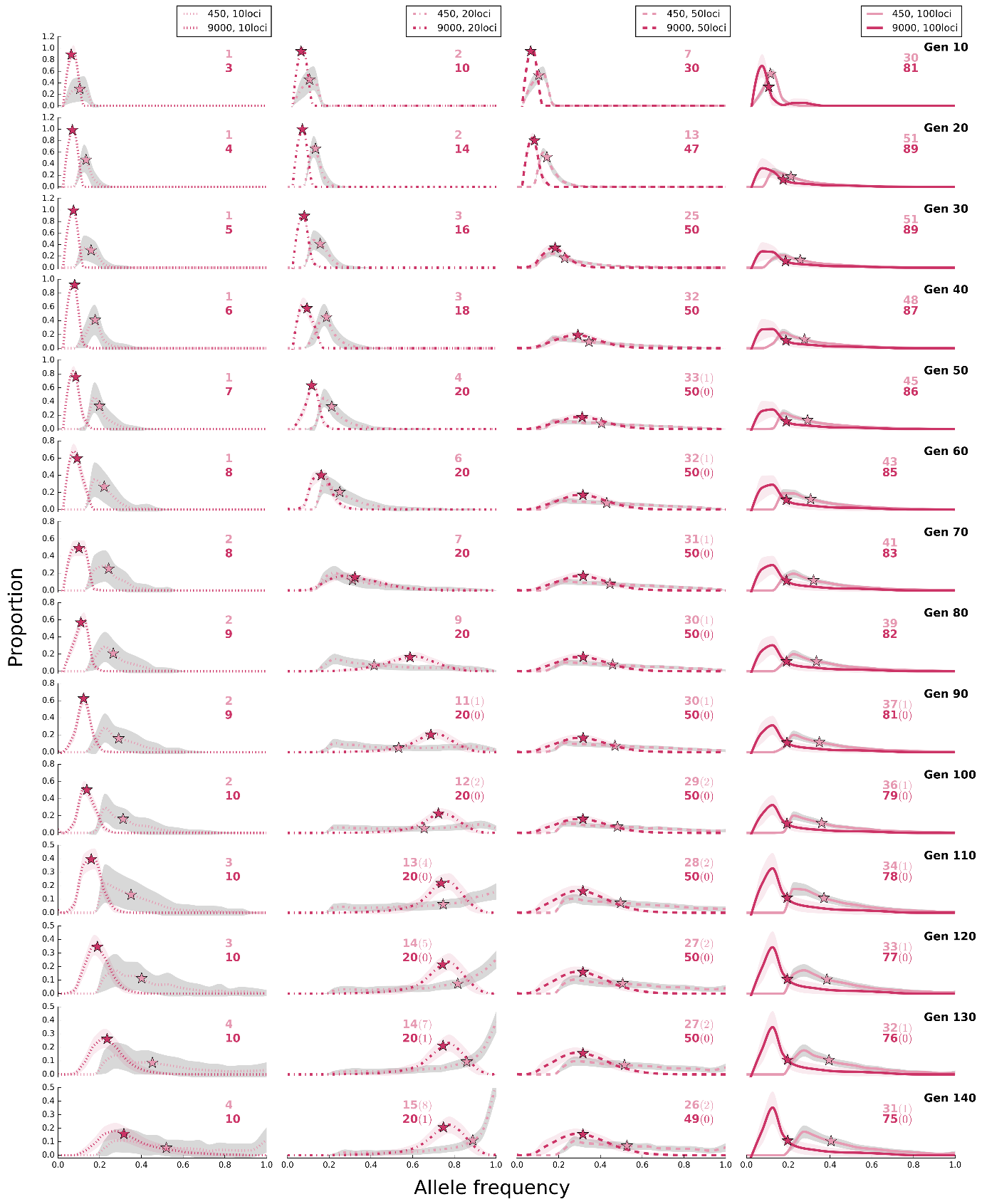
**

**Figure S7** Influence of population size and number of selection target on the site frequency spectrum of selected alleles for the trait optimum model. Populations with 450 and 9,000 individuals under trait optimum model are shown across 140 generations with 10 generation intervals (scenario B in Table 1b). The lines (dotted: 10, dash dotted: 20, dashed: 50, and solid: 100 loci) show the average (binned from 0 to 1 with 0.05 intervals) frequency of selected alleles across 500 replicates and shaded areas depict standard deviation. On the Y-axis (proportion) we show the fraction of loci that experienced a larger frequency increase than expected under neutrality. Asterisks depict the median frequency increase of selected alleles averaged across 500 replicates. The number of alleles with frequency increase averaged across 500 replicates is shown with colors corresponding to the labels. The number of alleles with sweep-like signature (frequency ≥ 0.9) averaged across 500 replicates, if present, is shown in parentheses. Rows correspond to time points of the experiments, i.e. generation, and shown as ‘Gen #’.

**
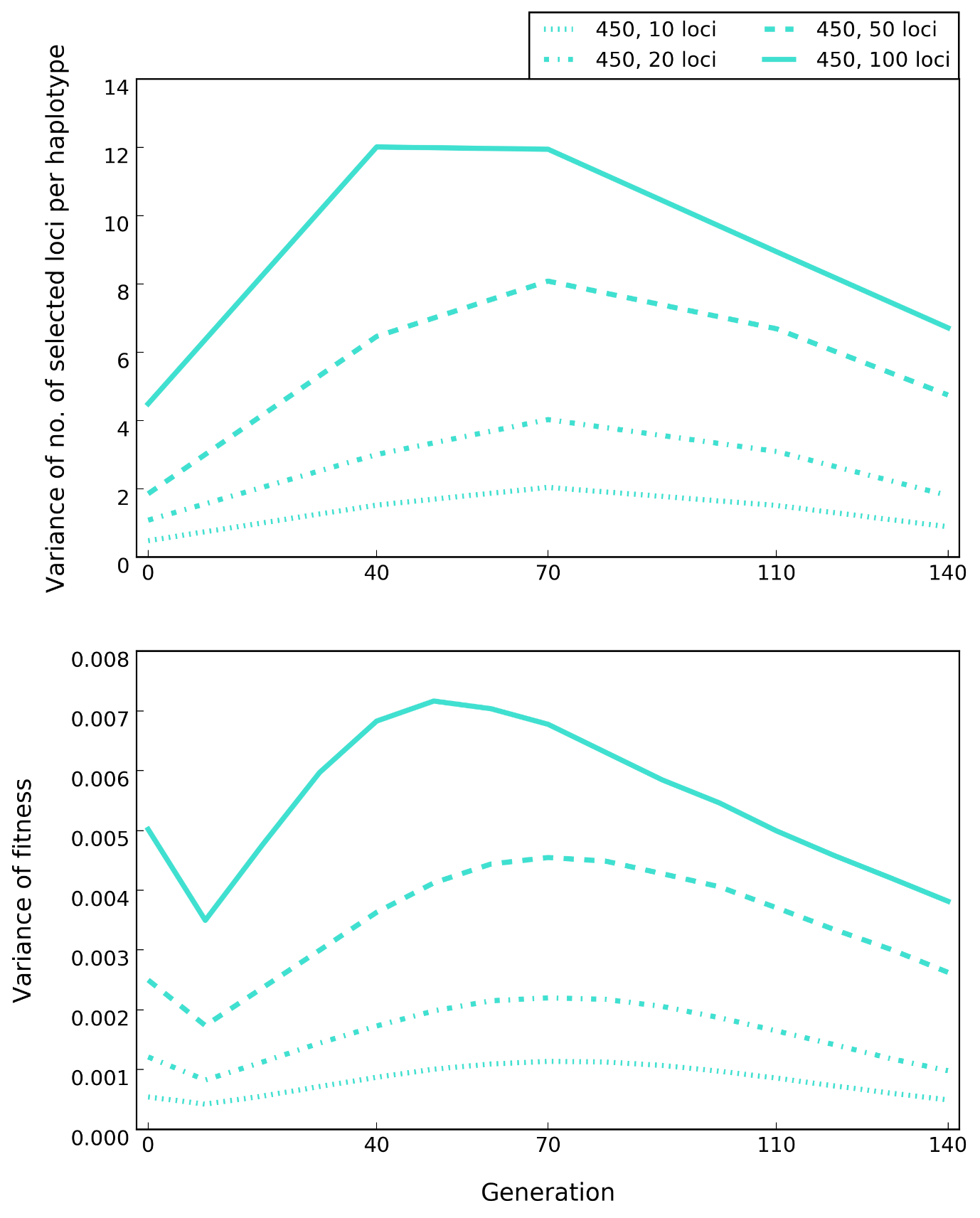
**

**Figure S8** Small populations (450 individuals) with more selected loci generate haplotypes with larger variance in the number of selected alleles and subsequently have higher variance in fitness under sweep model (scenario B in Table 1a). In the top panel, lines show the variance of the number of beneficial loci per haplotype (binned from 0 to 100 with intervals of 1) averaged across 50 replicates and the shaded areas show standard deviation. In the bottom panel, lines show variance of median fitness of the population across 500 replicates. Fitness is log_10_ transformed. Number of beneficial loci per haplotype for time points 0, 40, 70, 110 and 140 are shown, and fitness throughout 140 generations with intervals of 10 generations are shown.

**A**


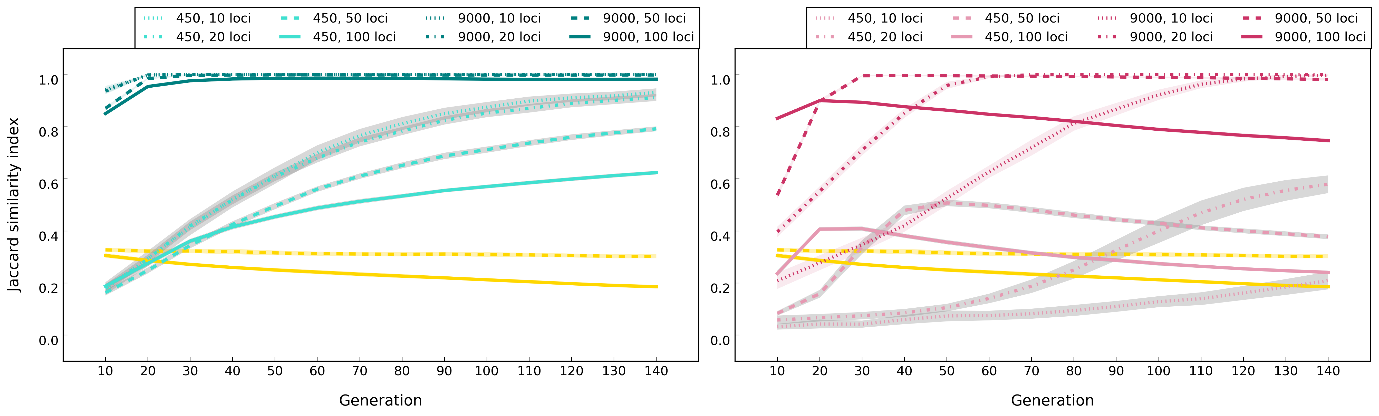


**B**

**
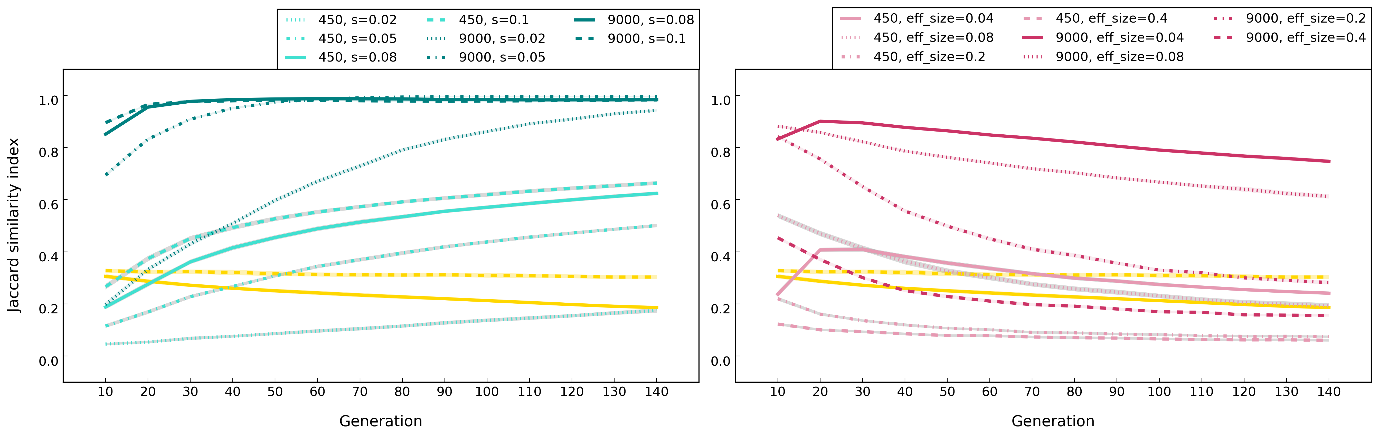
**

**Figure S9** Patterns of parallelism (i.e., similarity among replicate populations) under sweep and trait optimum models in populations of 450 and 9,000 individuals. We used the Jaccard similarity index, which quantifies the extent to which alleles are shared among replicates (0 = no overlap, 1 = complete sharing), to quantify the similarity among replicate populations. Jaccard similarity indices in populations with A) different number of beneficial loci (scenario B in Table 1a and 1b) and B) different effect sizes (scenario C in Table 1a and 1b) under sweep (left panels) and trait optimum (right panels) models. Lines show the average pairwise Jaccard indices among replicates for 50 sets of 10-replicate evolution experiments and the shaded areas show standard deviation. Solid and dotted yellow lines show the average Jaccard index under neutrality in populations of 450 and 9,000 individuals, respectively and standard deviation is shown as the shaded area around each line. For the trait optimum model, the generation the optimum phenotype is reached is shown in Fig. 5 (for A) and Fig. S11 (for B). *s*: selection coefficient, eff_size: effect size


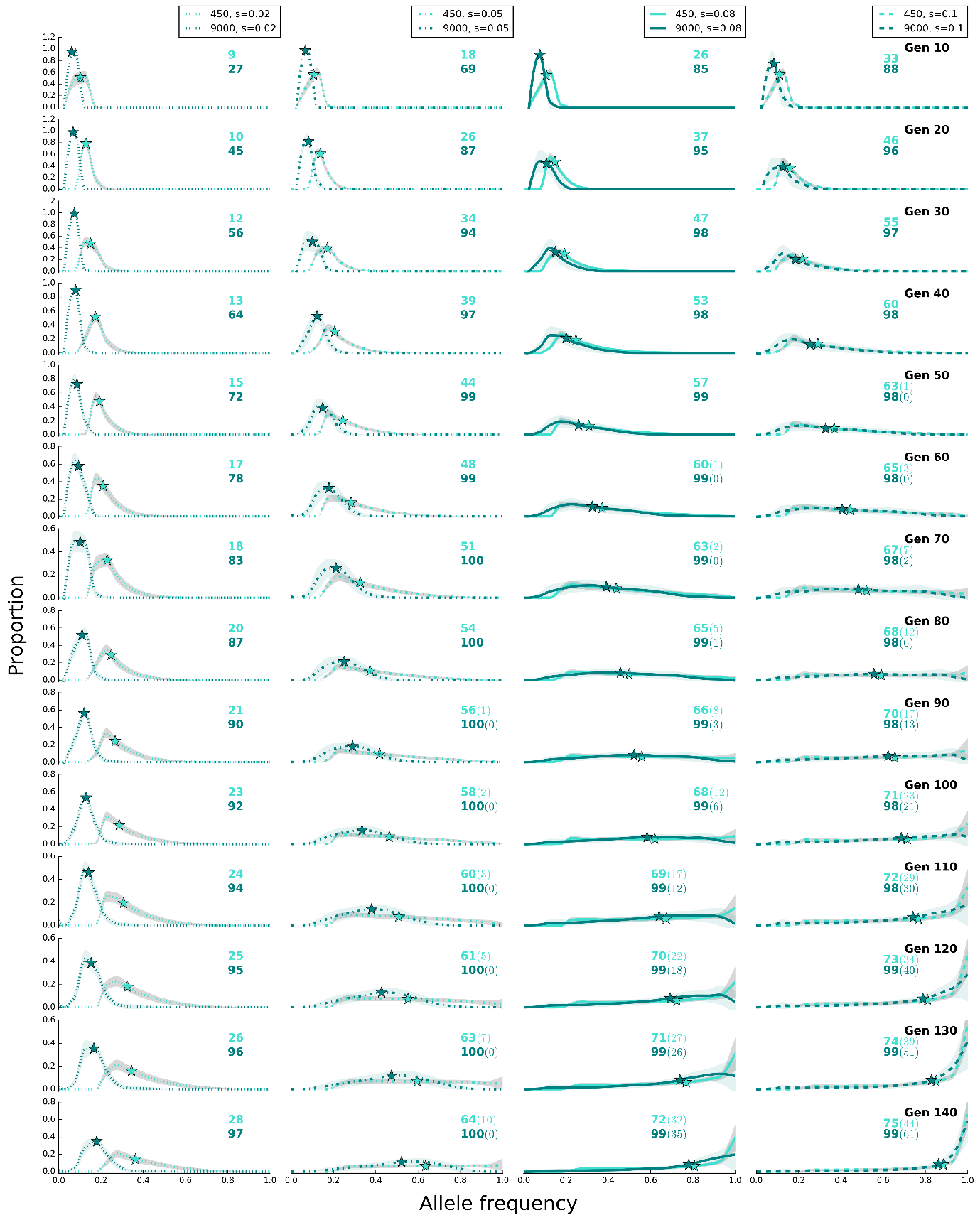


**Figure S10** Effect size determines the site frequency spectrum under sweep model. Populations with 450 and 9,000 individuals have a characteristic site frequency spectrum of selected alleles across 140 generations with 10 generation intervals (scenario C in Table 1b). The lines (dotted: s=0.02, dash dotted: s=0.05, solid: s=0.08, and dashed: s=0.1) show the average (binned from 0 to 1 with 0.05 intervals) frequency of selected alleles across 500 replicates and shaded areas depict standard deviation. On the Y-axis (proportion) we show the fraction of loci that experienced a larger frequency increase than expected under neutrality. Asterisks depict the median frequency increase of selected alleles averaged across 500 replicates. The number of alleles with frequency increase averaged across 500 replicates is shown with colors corresponding to the labels. The number of alleles with sweep-like signature (frequency ≥ 0.9) averaged across 500 replicates, if present, is shown in parentheses. Rows correspond to time points of the experiments, i.e. generation, and shown as ‘Gen #’.


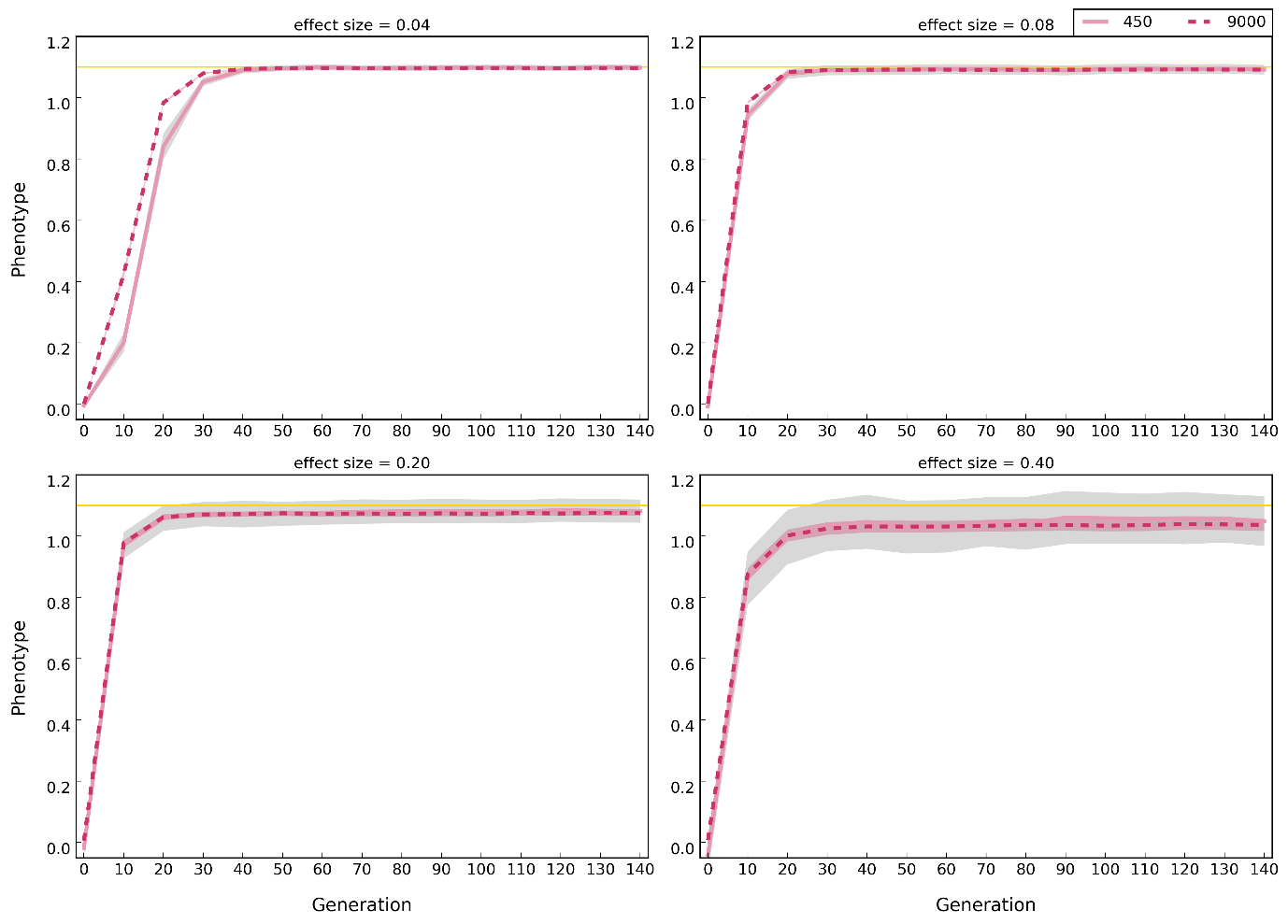


**Figure S11** The optimal phenotype in populations of 450 (solid lines) and 9,000 (dotted lines) individuals with loci of larger effect size is reached earlier under trait optimum model (scenario C in Table 1b). Lines show the median phenotype of populations averaged across 500 replicates and the shaded area around each line shows standard deviation. The optimum phenotype is 1.1 (shown by yellow lines). Note that the distance between the population phenotype at generation 0 and the optimum phenotype is equal across simulations with loci of different effect sizes. The plotted phenotype is normalized to account for different phenotypic means in the founder population; the mean phenotype of each population at F0 is subtracted from the phenotype of every individual at each time point.


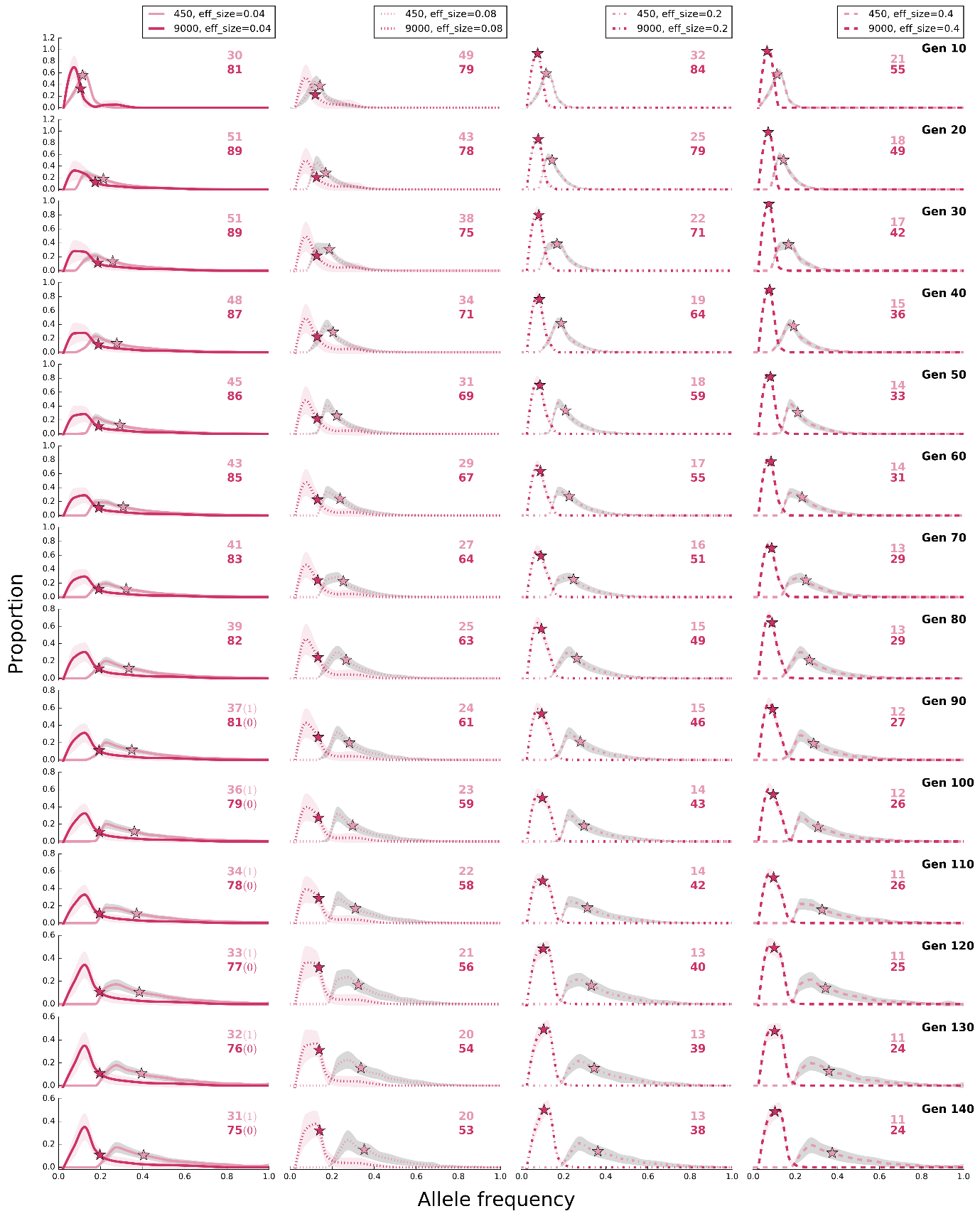


**Figure S12** The influence of effect size and population size on the site frequency spectrum of selected alleles under trait optimum model. Populations with 450 and 9,000 individuals under trait optimum model are shown across 140 generations with 10 generation intervals (scenario C in Table 1b). The lines (solid: 0.04, dotted: 0.08, dashed dotted: 0.2, and dashed: 0.4) show the average (binned from 0 to 1 with 0.05 intervals) frequency of selected alleles across 500 replicates and shaded areas depict standard deviation. On the Y-axis (proportion) we show the fraction of loci that experienced a larger frequency increase than expected under neutrality. Asterisks depict the median frequency increase of selected alleles averaged across 500 replicates. The number of alleles with frequency increase averaged across 500 replicates is shown with colors corresponding to the labels. The number of alleles with sweep-like signature (frequency ≥ 0.9) averaged across 500 replicates, if present, is shown in parentheses. Rows correspond to time points of the experiments, i.e. generation, and shown as ‘Gen #’.

**A**

**B**

**Figure S13** Guassian fitness function for the trait optimum model for A) different number of loci (scenario B in Table 1b), and B) different effect sizes (scenario C in Table 1b). The phenotype optimum is shifted so that the phenotype of the population regardless of the number of loci or their effect sizes is in equal distance from the optimum. Blue line depicts the starting phenotype of the population and the red line shows the optimum phenotype. **A:** from top to bottom (left to right) shows fitness functions for 100, 50, 20 and 10 loci, **B:** from top to bottom (left to right) shows fitness functions for 0.04, 0.08, 0.2 and 0.4 locus effect size.
